# Supplementary material for: Impact of electronic palliative care coordination systems (EPaCCS) on care at the end of life across multiple care sectors, in one clinical commissioning group area, in England: a realist evaluation protocol
Source: BMJ Open. 2020 Mar 31;10(3):e031153. doi: 10.1136/bmjopen-2019-031153 (PMC7170566; doi:10.1136/bmjopen-2019-031153)

BMJ Open is committed to open peer review. As part of this commitment we make the peer review history of every article we publish publicly available.

When an article is published we post the peer reviewers' comments and the authors' responses online. We also post the versions of the paper that were used during peer review. These are the versions that the peer review comments apply to.

The versions of the paper that follow are the versions that were submitted during the peer review process. They are not the versions of record or the final published versions. They should not be cited or distributed as the published version of this manuscript.

BMJ Open is an open access journal and the full, final, typeset and author-corrected version of record of the manuscript is available on our site with no access controls, subscription charges or pay-per-view fees (http://bmjopen.bmj.com).

If you have any questions on BMJ Open's open peer review process please email info.bmjopen@bmj.com

BMJ Open

THE IMPACT OF ELECTRONIC PALLIATIVE CARE COORDINATION SYSTEMS (EPaCCS) ON CARE AT THE END OF LIFE: A REALIST EVALUATION PROTOCOL

Journal:	*BMJ Open*
Manuscript ID	bmjopen-2019-031153
Article Type:	Protocol
Date Submitted by the Author:	07-May-2019
Complete List of Authors:	Pocock, Lucy; University of Bristol, Centre for Academic Primary Care<br>French, Lydia; University of Bristol, Centre for Academic Primary Care<br>Farr, Michelle; University of Bristol, NIHR CLAHRC West, Bristol Medical School<br>Morris, Richard; University of Bristol, School of Social & Community Medicine<br>Purdy, Sarah; University of Bristol, Centre for Academic Primary Care
Keywords:	PALLIATIVE CARE, Quality in health care < HEALTH SERVICES ADMINISTRATION & MANAGEMENT, PRIMARY CARE, QUALITATIVE RESEARCH

SCHOLARONE™
Manuscripts

I, the Submitting Author has the right to grant and does grant on behalf of all authors of the Work (as defined in the below author licence), an exclusive licence and/or a non-exclusive licence for contributions from authors who are: i) UK Crown employees; ii) where BMJ has agreed a CC-BY licence shall apply, and/or iii) in accordance with the terms applicable for US Federal Government officers or employees acting as part of their official duties; on a worldwide, perpetual, irrevocable, royalty-free basis to BMJ Publishing Group Ltd ("BMJ") its licensees and where the relevant Journal is co-owned by BMJ to the co-owners of the Journal, to publish the Work in this journal and any other BMJ products and to exploit all rights, as set out in our licence.

The Submitting Author accepts and understands that any supply made under these terms is made by BMJ to the Submitting Author unless you are acting as an employee on behalf of your employer or a postgraduate student of an affiliated institution which is paying any applicable article publishing charge ("APC") for Open Access articles. Where the Submitting Author wishes to make the Work available on an Open Access basis (and intends to pay the relevant APC), the terms of reuse of such Open Access shall be governed by a Creative Commons licence – details of these licences and which Creative Commons licence will apply to this Work are set out in our licence referred to above.

Other than as permitted in any relevant BMJ Author's Self Archiving Policies, I confirm this Work has not been accepted for publication elsewhere, is not being considered for publication elsewhere and does not duplicate material already published. I confirm all authors consent to publication of this Work and authorise the granting of this licence.

For peer review only

THE IMPACT OF ELECTRONIC PALLIATIVE CARE COORDINATION SYSTEMS (EPaCCS) ON CARE AT THE END OF LIFE: A REALIST EVALUATION PROTOCOL

Corresponding author

Lucy V Pocock, Population Health Sciences, Bristol Medical School, University of Bristol, Canynge Hall, 39 Whatley Road, Bristol, BS8 2PS

Lucy.pocock@bristol.ac.uk, Tel: 07810 002320

Co-authors

Lydia R M French, Population Health Sciences, Bristol Medical School, University of Bristol, Bristol, UK

Michelle Farr, The National Institute for Health Research Collaboration for Leadership in Applied Health Research and Care West (NIHR CLAHRC West) at University Hospitals Bristol NHS Foundation Trust, UK

Richard Morris, Population Health Sciences, Bristol Medical School, University of Bristol, Bristol, UK

Sarah Purdy, Population Health Sciences, Bristol Medical School, University of Bristol, Bristol, UK,

Word Count: 3862

For peer review only

Abstract

Introduction: Electronic palliative care coordination systems (EPaCCS) aim to support people approaching the end-of life to receive consistent care, according to their wishes, that is coordinated effectively across multiple care sectors. They are in use across the UK although empirical evidence into their effectiveness is poor. This paper presents a protocol of a mixed-methods study, to understand how, and by whom, EPaCCS are being used and whether EPaCCS are enabling Healthcare Professionals (HCPs) to coordinate patients' end-of-life (EOL) care.

Methods and Analysis: This is a mixed-methods study, carried out within a realist paradigm, to evaluate the impact of an EPaCCS on EOL care as provided by a Clinical Commissioning Group (CCG) in England. This study has two aims: 1. Describe the socio-demographic characteristics of patients who die with an EPaCCS record, their underlying cause of death and place of death and compare these with patients who die without an EPaCCS record. 2. Explore the impact of an EPaCCS on the experience of receiving EOL care for patients and their carers, and understand HCPs' views and experiences of utilising an EPaCCS to coordinate care for their patients. The study will be conducted in five phases: (1) development of the initial programme theory; (2) focus group with CCG stakeholder board; (3) individual interviews with HCPs, patients, current and bereaved carers; (4) retrospective cohort study of routinely collected data on EPaCCS usage and (5) data analysis and synthesis of study findings.

Ethics and dissemination: The study has been approved by NHS South West – Frenchay Research Ethics Committee (REC reference number: 18/SW/0198). Findings will be published in a wide range of outputs targeted at key audiences.

Strengths and limitations of this study

For peer review only

- The study will clearly articulate the preliminary theories and assumptions about EPaCCS and how they are expected to work.
- The study will test and refine these theories throughout the study using existing empirical evidence, qualitative data collected from healthcare professionals, patients and carers, and routinely collected quantitative data.
- The study will investigate the impact of only one of the many EPaCCS developed and implemented in the UK.

Key words

Palliative Care, Quality in Health Care, Primary Care, Qualitative Research

For peer review only

Background

People at the end-of-life (taken here to mean in the last 12 months of life) frequently receive care from a wide variety of teams and organisations. Much of this care is accessed in the out-of-hours period (overnight and at the weekend), when they are unlikely to see a Healthcare Professional (HCP) who knows them well. Out-of-hours provision of palliative care (defined by NICE as “the active holistic care of patients with advanced, progressive illness” (1)) has been identified as a key priority for future research by the Palliative and End of Life Care Priority Setting Partnership, initiated in 2013 by Marie Curie (2). This process identified the top 10 unanswered research questions and the question with the highest priority was, “What are the best ways of providing palliative care outside of working hours to avoid crises and help patients to stay in their place of choice?”

Continuity of care is important for anyone with complex health and social care needs, but particularly for those at the end-of-life (EOL) (3). A lack of information sharing has been repeatedly cited as a barrier to the provision of good quality EOL care outside of normal working hours (4,5,6). Recent studies looking at the experiences and needs of people seeking palliative care out-of-hours found that most patients expect the HCP to be able to access a summary of their complex medical history and many voiced concerns that their full record could not be accessed by out-of-hours clinicians (5,7). For some patients, lack of access to their notes is a deterrent to accessing care out-of-hours (6).

Nationally, the policy drive to address the issue of information continuity has resulted in the development of Electronic Palliative Care Coordination Systems, or EPaCCS (5,8,9,10). These records are usually completed by the patients’ GP or community nurse, including patients’ advance care wishes, and are accessible across multiple care sectors (10). The purpose of EPaCCS is to provide a shared local record for health and social care professionals, with key information about an individual approaching the EOL, including their

For peer review only

expressed preferences for care. In accordance with the Quality Statements in the NICE End of Life Care Standard for Adults (11) the intent is for EPaCCS to support people approaching the EOL to receive consistent care that is coordinated effectively across all relevant settings.

An EPaCCS record can take various forms, including web-based electronic registers, systems based on sharing care summaries or care plans, alongside patients' electronic records. They store a dynamic record of a patient's medical condition, treatment, wishes and preferences, and provide information about the medication a patient is receiving, contact details of any carers, and services involved in providing care and support to the patient.

Sharing information about patients' EOL care improves coordination and communication across care settings (12). It reduces unnecessary and unplanned hospital admissions, ambulance journeys, inappropriate interventions and use of unscheduled care, limits the likelihood of HCPs and patients having to repeat 'difficult conversations' and enables a patient to die in their preferred place (13).

It is now recognised that place of death is unlikely to be the most important factor in achieving a good death and a recent UK study has proposed that it is the presence of loved ones that is more important than the physical location (14). However, death in preferred place remains a significant measure of quality of death (15) and, according to the Voices survey of bereaved people, despite 81% of respondents believing that the deceased had wanted to die at home less than a quarter of people actually achieve this (16, 17).

Quantitative studies have shown striking differences in place of death with EPaCCS but are potentially biased and confounded (12). A more recent study challenges these assumptions, suggesting that the increase in home deaths could in fact be due to selection bias (few secondary care colleagues used the systems and therefore hospital deaths are not captured) (10). The findings of this study also underscore the importance of qualitative approaches,

For peer review only

which can offer crucial insights into what is happening on the ground, away from broad claims of EPaCCS benefits arrived at solely through quantitative methods. Without understanding the experiences of patients and carers, together with the perspectives of HCPs, it is difficult to evaluate the effectiveness of EPaCCS (18).

Technology in isolation is not guaranteed to bring benefit and the initiation of an EPaCCS relies on healthcare professionals initiating conversations about death and dying. There is evidence that these conversations are difficult for HCPs (19), with many choosing to avoid the conversation altogether (20). What impact EPaCCS has upon these conversations, if any, is not clear.

A recent independent evaluation of EPaCCS found that "it was not possible to demonstrate that EPaCCS was making any difference to the care patients were receiving at the end-of-life because the range of clients for whom EPaCCS was being used remains focused on cancer, and the ability of EPaCCS systems to report on progress and outcomes remains generally poor" (21).

We therefore do not know if EPaCCS acts to improve practice or whether it simply documents and reflects what is already taking place in practice (21). Indeed, the need to gather evidence of effectiveness of EPaCCS before widespread and uncritical adoption by the NHS is key.

To summarise, very little research has been carried out to understand how, and by whom, EPaCCS are being used and, perhaps more importantly, whether EPaCCS are enabling HCPs to support patients' EOL wishes. Rigorous evaluation and research are urgently needed to investigate to what extent EPaCCS influence services working together to support 'a good death', the outcome that stakeholders think is of most importance (22).

For peer review only

Aims, Objectives and Research Questions

The study has two aims. These are to:

1. Describe the socio-demographic characteristics of patients who die with an EPaCCS record, their underlying cause of death and place of death and compare these with patients who die without an EPaCCS record.

2. Explore the impact of an EPaCCS on the experience of receiving EOL care for patients and their carers, and understand HCPs' views and experiences of utilising an EPaCCS to provide palliative care to their patients.

Project methodology

Study setting

This study will be implemented in England, where one Clinical Commissioning Group (CCG) has recently developed and rolled out an EPaCCS. This involved the dissemination of an EMIS template, which was circulated to General Practices, to help ensure consistent data entry and coding. EMIS is the most widely used primary care clinical system in the UK and allows real-time patient information to be shared securely between different organisations. All practices in the CCG area are EMIS users and it is now also used by the local hospice and many of the community nursing teams. It is not, however, used routinely by secondary care organisations, the ambulance service, or providers of social care.

The EPaCCS template was developed following extensive local clinical consultation and the National Information Standard (23). Information from this template went live across the local health community, via the integrated digital care record used by health and social care professionals in the CCG area, at the end of February 2018.

Conceptual framework

For peer review only

This study will draw on a realist evaluation approach (24). A randomised, experimental study design is not possible as the implementation of EPaCCS has been strongly advocated and promoted by NHS England, with 83% of Clinical Commissioning Groups (CCGs) in England reported to have an operational EPaCCS, or be in the planning stages, by 2013 (25). The CCG had recently operationalised an EPaCCS at the time of study design.

By their nature, EPaCCS involve multiple organisations and a multidisciplinary style of work. It therefore requires a novel methodological approach to evaluation as described in this protocol.

Realist evaluation is a theory-driven approach designed for evaluating complex interventions, such as EPaCCS, where the outcomes are influenced by the way the intervention is delivered and in what context. Due to this complexity, any evaluation of EPaCCS seeking to determine linear causal relationships or simply find out if the intervention "works" is unlikely to be useful. Pawson and Tilley (24), the developers of realistic evaluation, suggest that the results of an intervention (outcomes) are dependent on the introduction of appropriate reasoning and resources (mechanisms) and how these then interact with existing social and cultural condition (contexts). For the purposes of this study, we have defined context (C), mechanism (M) and outcome (O) in Figure 1.

Figure 1 – Definition of Context, Mechanism and Outcome

A realist approach acknowledges that complex interventions only ever work for certain people, in particular circumstances. The key task of a realist evaluation is to understand and explain these patterns by asking the exploratory question: *what works, for whom and in what circumstances?*

According to Pawson, interventions or "programmes", such as the EPaCCS, are 'theories incarnate' and every programme has a theoretical underpinning (27). Before conducting a

For peer review only

realist evaluation, the researchers must develop a theory, or theories, that explain what works, for whom, under what circumstances and how. This is sometimes known as the "initial programme theory" and is described through CMO conjectures. This theory, or theories, are then tested through the process of the evaluation.

The study will be conducted in five phases: (1) development of the initial programme theory; (2) focus group with CCG stakeholder board; (3) individual interviews with healthcare professionals (HCPs), patients, current and bereaved carers; (4) retrospective cohort study of routinely collected data on EPaCCS usage and (5) data analysis and synthesis of study findings.

Phase one: development of the initial programme theory

Phase one is complete and included identification of relevant literature from electronic searches of databases. Reference lists of relevant papers were scanned, and citation searches conducted. Grey literature relating to policy and organisational-based material were sought by searching government and other specialist websites. The lead researcher's own experiences as a General Practitioner (GP) were used as "informed guesswork" (28) and initial meetings were held with key stakeholders, including patients at the local hospice and commissioners. These initial engagements were informal and were patient and public involvement activities (detailed further below). They did not constitute formal interviews requiring ethics clearance.

At an early stage of the programme theory development, the CMO conjectures were reviewed by, and discussed with, Dr Justin Jagosh, Director of the Centre for Advancement in Realist Evaluation and Synthesis.

For peer review only

The initial programme theory forms a set of hypotheses on what the mechanisms may be, what groups may benefit most or least and the contextual factors that might be important to its success or failure. These hypotheses will be interrogated and tested in phases 2-5 of the study.

Figure 2 - Visual representation of the initial programme theory at a macro level.

Phase two: focus group with CCG stakeholder board

The CCG EOL care board is a multi-disciplinary, multi-organisational system board, whose members are high-level stakeholder representatives from across the CCG, including representatives from community nursing teams, primary care, the ambulance service, local hospices, care homes and secondary care. The purpose of the board is to review the current commissioning across all geographical provision, ensuring unified pathways for community, primary and secondary providers to provide consistency for all patients, carers and staff across the system.

Board members will be invited to take part in a focus group following their attendance at the monthly board meeting. Focus groups allow for social interaction, which can help to reveal issues and subsequent points of view that may not be prompted or discovered through individual interviews. This approach will help the study team to gain as wide an understanding as possible of the process of commissioning the EPaCCS and help to refine the initial programme theory.

Participants will be consented to take part in the study prior to the focus group by either LF or LP who will be facilitating. A topic guide will be used to steer the focus group and will enable the research team to test and refine the initial programme theory prior to commencing the in-depth interviews (see table 1).

For peer review only

It is anticipated that the focus group will last approximately 45 minutes and that approximately 5-10 participants will take part in it. The focus group will be audio taped and transcribed verbatim.

Table 1: Programme theory for the EPaCCs study, comprising the 17 context, mechanism and outcome (CMO) statements that inform the programme theory, and the questions that will be used, in the focus group with the end-of-life Board, to investigate each CMO statement.

EPaCCs Process	CMOs	Focus group questions
**Commissioning**	1. If the strategy behind the EPaCCs is definable, deliverable and measureable, the aim, purpose and outcomes of EPaCCS will be clear. ***(strategy)***	• How will you evaluate EPaCCS success?<br>• What are the markers of success for you?<br>• What is the CCGs long term vision for the EPaCCS?
	2. If HCPs engage with the EPaCCS positively on early usage and see it as an improvement on any previous EOL register, HCPs will engage positively with EPaCCS. ***(engagement)***	• Given that the previous EOL register was generally not well thought of, or used, how did the CCG plan to get HCPS on board?<br>• How do you think the EPaCCS has been received?
**Commissioning/Uptake/Adoption**	3. If the EPaCCS is well-publicised and marketed to all stakeholders HCPs will be aware of EPaCCS, understand the aim and purpose of the EPaCCS, and will initiate an EPaCCs template and/or access an EPaCCS record. ***(publicity)***	• How was the EPaCCS publicised and marketed to different groups of healthcare professionals (HCPS)? What are your views on how effective this has been?<br>• How aware do you think HCPs are of EPaCCS and do you think they understand its purpose and importance?
	4. If HCPs receive sufficient support and training, so that they know how to use it, they and will initiate an EPaCCs template and/or access an EPaCCS record. ***(training)***	• Can you tell us about the CCG strategy for providing training and support to different groups of HCPs in the EPaCCS rollout?<br>• What do you think about this, and how effective it has been?
**Uptake/Adoption**	5. If HCPs have the time and/or resources to learn a new system, an EPaCCS template will be initiated. ***(time & resources)***	• There are a significant number of GP practices that have not initiated an EPaCCS – do you have any thoughts about why this might be?<br>• Do you think all HCPs will have the time and resources (i.e. they are connected to a computer, have internet and NHS network access) to learn and new system and access EPaCCS?
	6. If HCPs are incentivised to use EPaCCS, an EPaCCS template will be initiated. ***(incentives)***	• Do HCPs have other ways of obtaining the information contained on EPaCCs?<br>• What might these be, and are these ways better or worse, more reliable or less reliable?
**Uptake**	7. If the patient consents to information-sharing and storage of information about their care preferences, an EPaCCS template will be initiated. ***(information sharing)***	• For the EPaCCS to be effective, patients must consent to information-sharing, and the storage of information.<br>• Did you anticipate that this would raise any issues?
	8. If HCPs are near to a computer, are connected to the internet and have access to the GP EMIS record, an EPaCCS template will be initiated. ***(access to system)***	• There is a theory that because EPaCCS is an electronic record, presently only updateable by the GP on EMIS, that this will have an impact on the ability of others to access it and update it and own it. Do you see this as an issue?<br>• What impact do you think this might present?
	9. If HCPs feel able/comfortable having advanced care planning	• How do you think HCPs feel about having ACP conversations with patients?

	(ACP) conversations with patients, an EPaCCS template will be initiated. ***(ACP conversations)***	• Research suggests that patients with non-malignant diagnoses are less likely to be added to EPaCCS.<br>• Do you think this is the case and if so why? Are there other patient groups who might be underrepresented on the EPaCCS?
	10. If HCPs feel that the EPaCCS facilitates, potentially difficult, ACP conversations an EPaCCS template will be initiated. ***(ACP conversations)***	• Some would argue that the EPaCCS template might facilitate ACP conversations with patients – what are your thoughts on this?
	11. If the patient is willing, and has capacity to have ACP conversations, an EPaCCS template will be initiated. ***(ACP conversations)***	• Patients can only record their wishes if they are able to have a conversation with an HCP – what issues do you think this might present?
**Adoption**	12. If EOLC information about a patient can be accessed more efficiently in other ways (i.e. speaking with carer or reading other sources of information) the information on the EPaCCS template may not be accessed. ***(single point of access)***	• Are there any other sources of information that HCPs might access to establish the EOL wishes and needs of a patient and do you think they present an issue of the uptake of EPaCCS?
	13. If HCPs are near to a computer, are connected to the internet and have access to the NHS Network an EPaCCs template will be accessed. ***(access to system)***	• There is a theory that because EPaCCS is an electronic record, presently only updateable by the GP on EMIS, that this will have an impact on the ability of others to access it and update it and own it.<br>• Do you see this as an issue? What impact do you think this might present?
**Adoption/Care Coordination**	14. If the information does not reflect the current wishes of the patient, care may not be aligned with the patients' preferences. ***(patient preferences)***	• Do you feel that the EPaCCs adequately reflects patient's wishes and preferences for care?
	15. If the patient does not have clear or clinically meetable preferences, or their wishes are subject to frequent change, care may not be aligned with the patient's wishes. ***(patient preferences)***	• Do you feel the EPaCCS adequately reflects the patient's/carer's wishes and preferences regarding end of life care and do you feel these wishes are deliverable? If not, why might this be and what needs to be improved?
**Care Coordination**	16. If HCPs access EPaCCS and consider the information contained within it to be trustworthy (current, relevant, detailed and useful) care will be coordinated by EPaCCs and this care will align with the patient's wishes. ***(trustworthiness of EPaCCS)***	• Do you think the EPaCCS contains all the information HCPs need to enact their patient's wishes and coordinate their patient's care?<br>• Do you consider it to be current, relevant, detailed and useful? If not, why might this be and what needs to be improved?
	17. If EPaCCS does not enhance or improve the care that is already being delivered care may not be coordinated by EPaCCS, consistent or reflect the patients' preferences. ***(patient outcomes)***	• What are your thoughts on the notion that: 'The EPaCCS is not coordinating care, it is simply recording what is already being done'

Phase three: individual interviews with healthcare professionals (HCPs), patients, current and bereaved carers

Healthcare professionals (HCPs)

HCPs from community nursing teams, primary care, the ambulance service, the local hospices, care homes and secondary care will be invited to participate.

GPs working in practices within the CCG area will be invited to support the study and the study team will purposively sample from a list of practices, to include practices that are high-users of EPaCCS and low users of EPaCCS based on data compiled by the CCG.

The research team will purposively sample HCPs who express an interest in participating according to gender, age, and profession to ensure maximum variation in the sample. All interviews with HCPs will take place over the telephone for both pragmatic and methodological reasons. Conducting interviews over the telephone will reduce the time and cost to the study that may be involved in travelling to interviews and well-planned telephone interviews can gather the same material as those held face to face (29).

A topic guide, informed by the evolving programme theory, will be used to ensure consistency across the interviews. This will enable the research team to compare the views of each group at the stage of data analysis. Interviews will last approximately 30 minutes and it is anticipated that approximately 3-5 HCPs will be interviewed from each group (18-30 in total).

Patients, current and bereaved carers

Patients will be approached to take part in interviews through their GP surgeries or the local hospice. The research team will purposively sample from a list of practices, to include practices that are high-users of EPaCCS and low users of EPaCCS based on data compiled by the CCG. Selected practices will be asked to identify patients, aged 18 and over, receiving EOL care, who they consider might be eligible to take part in the study.

Inclusion criteria:

For peer review only

1. Capacity to give informed consent
2. Aged 18 and over
3. Prognosis 12 months or less (patient aware of this prognosis)

Potential participants will be sampled purposively to include patients across the age range, with and without an EPaCCS record, with both malignant and non-malignant health conditions.

To recruit patients to the study from the local hospice we will liaise with key clinical staff, who will be responsible for identifying appropriate patients. Once again, potential participants will be sampled purposively to include patients across the age range, with and without an EPaCCS record, with both malignant and non-malignant health conditions.

Alongside their own study information pack, all patients will be given a carer information pack which they can choose to give to their carer if they are happy for their carer to participate in the study.

GPs will also be asked to identify recently bereaved carers (between 8 weeks and 6 months of the death of their relative), who they consider might be eligible to take part in the study. GPs will be sent details of how to perform an appropriate search within EMIS to identify potential participants.

Interviews with carers will be conducted one-to-one with the interviewer. Interviews with patients will be conducted either one-to-one with the interviewer or in the presence of their carer, according to the wishes of the patient.

Interviews will last approximately 45 minutes and it is anticipated that approximately 15 patients will be interviewed (10 EPaCCS patients and 5 non-EPaCCS patients) and 10 carers (to include both current and bereaved). All interviews will be audio taped and transcribed verbatim.

For peer review only

Phase four: retrospective cohort study of routinely collected data on EPaCCS usage

EMIS data will be accessed to identify all patients, aged 18 and over, who die in the CCG area between 22nd February 2018 and 21st February 2019. Patients will be identified as either having an EPaCCS record (EPaCCS patient) or not (non-EPaCCS patient).

EMIS will be used to characterise both EPaCCS and non-EPaCCS patients in terms of their gender, ethnicity and postcode (as a proxy for socio-economic status according to their Index of Multiple Deprivation Score). Data will be extracted to describe:

1. The proportion of patients that die with an EPaCCS record.
2. When the EPaCCS record is initiated (i.e. how many months/days prior to the patient's death), and by whom.
3. How frequently the EPaCCS record is updated once opened, and who makes any changes.
4. The underlying cause and place of death for EPaCCS and non-EPaCCS patients.
5. The number of hospital admissions and Emergency Department attendances for EPaCCS and non-EPaCCS patients in the last 12 months of life.

Data from the integrated digital care record will be accessed to describe which HCPs (GPs, community nurses, hospice HCPs, ambulance HCPs and secondary care clinicians) are accessing these shared end-of-life care records.

Phase five: data analysis and synthesis of study findings

Quantitative methods

For peer review only

Quantitative data will be analysed using Stata v15 and reported using descriptive statistics. Logistic regression will be used to determine the adjusted odds ratio and 95% confidence intervals for the associations between having an EPaCCS record and dying at home, adjusted for socio-demographic and medical characteristics (age, sex, deprivation and underlying cause of death).

Of approximately 8,000 deaths occurring in the CCG area over the year of study, we expect around 10% (800 deaths) of patients to have an EPaCCS. If the proportion of deaths occurring at home is expected to be 25% among those without an EPaCCS, we would have over 99% power to detect an absolute increase of 10% to 35% among those with an EPaCCS. The power would be about 84% if the proportion were increased by 5% to 30%.

Qualitative methods

Data analysis will be conducted using a realist approach informed by Jackson and Kolla's realistic evaluation analysis method (30). This analytic process will involve the following steps:

1. Coding individual units (a discrete C, M or O) within the narratives of the interviews.
2. Identifying the complex connections that link these codes together into dyads or triads.
3. Subsuming the linked codes into themes using thematic analysis (30).

Analysis will begin shortly after data collection starts and be ongoing and iterative. Analysis will inform further data collection: for instance, analytic insights from data gathered in earlier interviews will be used to develop and adapt the programme theory and in turn, identify any changes that need to be made to the topic guide for use during later interviews. The study will generate new programme theories to explain how the EPaCCS works, for whom, and any contextual influences and constraining factors that affect their initiation and usage. Emerging

analysis and findings will be discussed with PPI representatives, to explore and clarify findings.

Qualitative and quantitative data will be collected concurrently, giving equal weight to each (31). Data will be triangulated in order to test and refine the programme theories, accepting that any findings are fallible and with time and further study new data are bound to emerge (32). The synthesised study findings will establish the potential outcomes of EPaCCS, identify the underlying mechanisms which explain how they produce these effects and highlight the key contextual factors that affect their success or failure. Recommendations can then be made for the development and implementation of EPaCCS.

Patient and Public Involvement

To support the development of this study protocol, members of the study team (LF and LP) presented and discussed an outline proposal of this study to patients, staff and carers at the local hospice on two separate occasions in April 2018. Approximately 10 participants voluntarily took part in these discussions. These meetings raised several important issues which have been incorporated into the design of this study. Such issues included allowing patients the choice of whether to have a carer sit alongside them during their interview and which HCPs they felt it was important that the study team spoke to, due to the involvement they had in providing care for patients. The meetings also discussed what terms, wording and questions would be acceptable to patients and carers to read and hear in the study information documents and interviews.

At the end of both meetings, patients and carers were invited to continue to support the design of the study should they wished. Two members came forward expressing a wish to be more actively involved in the study. They have kindly been involved in reviewing all the study literature, including the topic guides, study information sheets and the lay summary for

For peer review only

this protocol. It is hoped that they will wish to continue their involvement with the study. This will include informing the content of materials for lay audiences, drawing links to groups and forums that the research team may be unaware of, and supporting the study team with the interpretation and dissemination of study findings. To ensure ongoing PPI, all patients and carers taking part in the study will be invited to support the ongoing development of the study.

Ethics and dissemination

The study has been approved by NHS South West – Frenchay Research Ethics Committee (REC reference number: 18/SW/0198).

The research team will disseminate the findings to a range of stakeholders. We will draw on the networks and expertise of the local CCG end-of-life care board to disseminate the research outputs widely and appropriately. Key audiences include patient and carer organisations, GPs and community nursing teams in primary care, ambulatory services and care home staff, HCPs working in secondary palliative care services and hospices, managers and directors within healthcare organisations with responsibility to provide high-quality services within budget and healthcare policymakers, nationally and internationally.

Acknowledgements

We would like to thank all the patients, carers and HCPs who helped to support the PPI in development of this study.

We would also like to thank Dr Justin Jagosh, Director of the Centre for Advancement in Realist Evaluation and Synthesis (https://realistmethodology-cares.org/), for his support and guidance in developing the initial programme theory.

For peer review only

Contributors

LP was the principal investigator for this qualitative study. LF, MF, RM and SP all contributed to the development of the study protocol. LP and LF drafted this manuscript. All authors contributed to the editing of the final manuscript and the refining of its intellectual content.

Funding

LP's time is paid for by a NIHR School for Primary Care Research GP Career Progression Fellowship. This fellowship funding will also meet the research costs. LF's time is paid for by NIHR Research Capability Funding. MF's time is supported by the NIHR Collaboration for Leadership in Applied Health Research and Care West (NIHR CLAHRC West). The study is supported by the NIHR West of England Clinical Research Network. The views expressed in this publication are those of the authors and not necessarily those of the NHS, the National Institute for Health Research or the Department of Health and Social Care.

Competing interests

We have read and understood BMJ policy on declaration of interests and declare that we have no competing interests.

References

1. National Institute for Health and Care Excellence (2004). Improving supportive and palliative care for adults with cancer. Cancer service guideline [CSG4].
2. Palliative and end of life care Priority Setting Partnership: Putting patients, carers and clinicians at the heart of palliative and end of life care research. January 2015.

For peer review only

Available at: https://www.mariecurie.org.uk/globalassets/media/documents/research/PeolcPSP_Final_Report.pdf (accessed 30/05/18)

3. Barker I, Steventon A, Deeny SR. Association between continuity of care in general practice and hospital admissions for ambulatory care sensitive conditions: cross sectional study of routinely collected, person level data. *BMJ* 2017;356:j84
4. Schweitzer B, Blankenstein N, Deliens L, Van Der Horst H. Out-of-hours palliative care provided by GP co-operatives in the Netherlands: A focus group study. *European Journal of General Practice*, 2011; 17: 160–166.
5. Hall S, Murchie P, Campbell C and Murray SA. Introducing an electronic Palliative Care Summary (ePCS) in Scotland: patient, carer and professional perspectives. *Family Practice* 2012; 29:576–585.
6. Richards SH, Winder R, Seamark C, Seamark D, Avery S. Gilbert J, Barwick A and Campbell JL. 2011: The experiences and needs of people seeking palliative health care out-of-hours: a qualitative study. *Primary Health Care Research & Development* 12(2), 165–78; available from: PM:21457601.
7. Leydon GM, Shergill NK, Campion-Smith C, et al. Discontinuity of care at end of life: a qualitative exploration of OOH end of life care. *BMJ Supportive & Palliative Care* 2013;3:412-421.
8. Purdy S, Lasseter G, Griffin T, Wye L. Impact of the Marie Curie Cancer Care Delivering Choice Programme in Somerset and North Somerset on place of death and hospital usage: a retrospective cohort study. *BMJ Supportive & Palliative Care* 2014;0:1–6.
9. Asprey A, Richards SH, Wright C, Seamark C, Seamark D and Moxon J. Transferring information to an out-of-hours primary care service for patients with palliative care

For peer review only

needs: an action research study to improve the use of handover forms. *Primary Health Care Research & Development* 2013; 14: 7–20.

10. Wye L, Lasseter G, Simmonds B, Duncan L, Percival J and Purdy S. Electronic palliative care coordinating systems (EPaCCS) may not facilitate home deaths: A mixed methods evaluation of end of life care in two English counties. *Journal of Research in Nursing*, 2016, Vol. 21(2) 96–107
11. National Institute for Health and Clinical Excellence (2011) End of Life Care for Adults. NICE Quality Standard [QS13]
12. Petrova M, Riley J, Abel J and Barclay S. Crash course in EPaCCS (Electronic Palliative Care Coordination Systems): 8 years of successes and failures in patient data sharing to learn from. *BMJ Supportive & Palliative Care* 2016;0:1–9.
13. Department of Health (2012) End of Life Care Strategy: Fourth Annual Report. Available at: https://assets.publishing.service.gov.uk/government/uploads/system/uploads/attachment_data/file/136486/End-of-Life-Care-Strategy-Fourth-Annual-report-web-version-v2.pdf (accessed 30/05/18)
14. Turner V, Flemming K. Socioeconomic factors affecting access to preferred place of death: A qualitative evidence synthesis. *Palliative medicine* 2019 Mar 8:0269216319835146.
15. Department of Health (2016) Our Commitment to you for end of life care: the Government Response to the Review of Choice in End of Life Care. Available at: https://assets.publishing.service.gov.uk/government/uploads/system/uploads/attachment_data/file/536326/choice-response.pdf (accessed 21/03/19)
16. Office for National Statistics. National Survey of Bereaved People (VOICES): England, 2015. Available at:

https://www.ons.gov.uk/peoplepopulationandcommunity/healthandsocialcare/healthcaresystem/bulletins/nationalsurveyofbereavedpeoplevoices/england2015#preferences-and-choice-at-the-end-of-life (accessed 30/05/18)

17. Public Health England, End of Life Care Profiles. Available at: https://fingertips.phe.org.uk/profile/end-of-life (accessed 30/05/18)
18. Allsop MJ, Kite S, McDermott S, Penn N, Millares-Martin P and Bennett MI. Electronic palliative care coordination systems: Devising and testing a methodology for evaluating documentation, *Palliative Medicine* 2017 May; 31(5): 475–482.
19. Fallowfield L and Jenkins V. Communicating sad, bad, and difficult news in medicine. *The Lancet*, 2004;*363*(9405), 312-319.
20. Gibbins J, McCoubrie R, and Forbes K. Why are newly qualified doctors unprepared to care for patients at the end of life? *Medical education*;2011, *45*(4), 389-399.
21. Whole Systems Partnership. (2016) *Independent evaluation of Electronic Palliative Care Co-ordination Systems (EPaCCS) in England: Final Report*, March 2016
22. Wye L, Lasseter GM, Percival JF, Simmonds BAJ, Duncan LJ, Purdy S. Independent Evaluation of the Marie Curie Cancer Care Delivering Choice Programme in Somerset and North Somerset. Centre for Primary Health Care, Department of Social and Community Medicine, The University of Bristol, 2012.
23. NHS Digital (2015). SCCI1580: Palliative Care Co-ordination: Core Content. Available at: https://digital.nhs.uk/data-and-information/information-standards/information-standards-and-data-collections-including-extractions/publications-and-notifications/standards-and-collections/scci1580-palliative-care-co-ordination-core-content (accessed 23/01/19)
24. Pawson R and Tilley N. Realistic Evaluation. London: Sage, 2005.

For peer review only

25. National End of Life Care Intelligence Network. (2014). *Electronic Palliative Care Co-ordination Systems (EPaCCS) in England: Survey of clinical commissioning groups (2013).*

26. Jagosh J, Bush PL, Salsberg J, Macaulay AC, Greenhalgh T, Wong G et al. A realist evaluation of community-based participatory research: partnership synergy, trust building and related ripple effects. BMC public health. 2015 Dec;15(1):725.

27. Pawson R. Evidence based policy: A realist perspective. London: Sage, 2006.

28. Greenhalgh T, Wong G, Jagosh J, et al Protocol—the RAMESES II study: developing guidance and reporting standards for realist evaluation BMJ Open 2015;5:e008567. doi: 10.1136/bmjopen-2015-008567

29. Sturges JE, Hanrahan KJ. Comparing telephone and face-to-face qualitative interviewing: a research note. Qual Res. 2004 Apr;4(1):107-18.

30. Jackson SF, Kolla G. A new realistic evaluation analysis method: linked coding of context, mechanism, and outcome relationships. American Journal of Evaluation. 2012 Sep;33(3):339-49.

31. Hanson WE, Creswell JW, Clark VL, Petska KS, Creswell JD. Mixed methods research designs in counseling psychology. Journal of counseling psychology. 2005 Apr;52(2):224.

32. Emmel N, Greenhalgh J, Manzano A, Monaghan M, Dalkin S (Eds). Doing realist research. London: Sage, 2018.

For peer review only

Figure 1 – Definition of Context, Mechanism and Outcome

Context

Something that existed prior to the introduction of the EPaCCS, for example cultural views and beliefs around talking about death and dying and Advanced Care Planning (ACP) conversations. Contexts might also refer to the setting, (e.g. primary or secondary care) or patient characteristics (e.g. underlying diagnosis, socio-demographics, mental capacity).

Mechanism

The intended or unintended resources created by an intervention and the response to those resources (cognitive, emotional, motivational etc) by Healthcare Professionals (HCPs), patients and carers. Mechanisms can pertain to why HCPs and patients choose (or choose not) to utilise the EPaCCS.

Outcome

An outcome will define the result of the EPaCCS whether intended (did the project succeed against the criteria it set itself at the outset), and also the unplanned and/or unexpected impacts.

Informed by (24) and (26).

For peer review only

Figure 2 – Visual representation of the initial programme theory at a macro level. See table 1 for associated CMO statements.

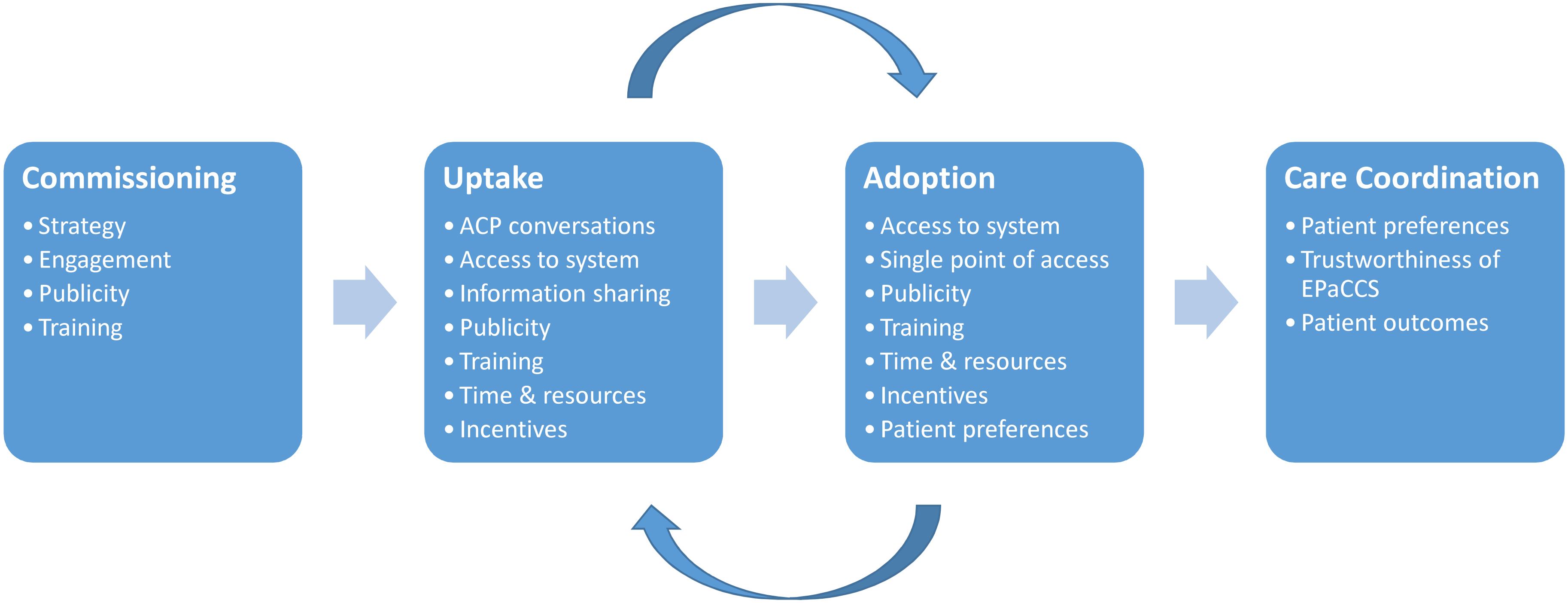

BMJ Open

THE IMPACT OF ELECTRONIC PALLIATIVE CARE COORDINATION SYSTEMS (EPaCCS) ON CARE AT THE END OF LIFE ACROSS MULTIPLE CARE SECTORS IN ENGLAND: A REALIST EVALUATION PROTOCOL

Journal:	*BMJ Open*
Manuscript ID	bmjopen-2019-031153.R1
Article Type:	Protocol
Date Submitted by the Author:	09-Aug-2019
Complete List of Authors:	Pocock, Lucy; University of Bristol, Centre for Academic Primary Care<br>French, Lydia; University of Bristol, Centre for Academic Primary Care<br>Farr, Michelle; University of Bristol, NIHR CLAHRC West, Bristol Medical School<br>Morris, Richard; University of Bristol, School of Social & Community Medicine<br>Purdy, Sarah; University of Bristol, Centre for Academic Primary Care
**Primary Subject Heading**:	Palliative care
Secondary Subject Heading:	Health services research
Keywords:	PALLIATIVE CARE, Quality in health care < HEALTH SERVICES ADMINISTRATION & MANAGEMENT, PRIMARY CARE, QUALITATIVE RESEARCH

SCHOLARONE™
Manuscripts

BMJ

I, the Submitting Author has the right to grant and does grant on behalf of all authors of the Work (as defined in the below author licence), an exclusive licence and/or a non-exclusive licence for contributions from authors who are: i) UK Crown employees; ii) where BMJ has agreed a CC-BY licence shall apply, and/or iii) in accordance with the terms applicable for US Federal Government officers or employees acting as part of their official duties; on a worldwide, perpetual, irrevocable, royalty-free basis to BMJ Publishing Group Ltd ("BMJ") its licensees and where the relevant Journal is co-owned by BMJ to the co-owners of the Journal, to publish the Work in this journal and any other BMJ products and to exploit all rights, as set out in our licence.

The Submitting Author accepts and understands that any supply made under these terms is made by BMJ to the Submitting Author unless you are acting as an employee on behalf of your employer or a postgraduate student of an affiliated institution which is paying any applicable article publishing charge ("APC") for Open Access articles. Where the Submitting Author wishes to make the Work available on an Open Access basis (and intends to pay the relevant APC), the terms of reuse of such Open Access shall be governed by a Creative Commons licence – details of these licences and which Creative Commons licence will apply to this Work are set out in our licence referred to above.

Other than as permitted in any relevant BMJ Author's Self Archiving Policies, I confirm this Work has not been accepted for publication elsewhere, is not being considered for publication elsewhere and does not duplicate material already published. I confirm all authors consent to publication of this Work and authorise the granting of this licence.

For peer review only

THE IMPACT OF ELECTRONIC PALLIATIVE CARE COORDINATION SYSTEMS (EPaCCS) ON CARE AT THE END OF LIFE ACROSS MULTIPLE CARE SECTORS IN ENGLAND: A REALIST EVALUATION PROTOCOL

Corresponding author

Lucy V Pocock, Population Health Sciences, Bristol Medical School, University of Bristol, Canynge Hall, 39 Whatley Road, Bristol, BS8 2PS

Lucy.pocock@bristol.ac.uk, Tel: 07810 002320

Co-authors

Lydia R M French, Population Health Sciences, Bristol Medical School, University of Bristol, Bristol, UK

Michelle Farr, The National Institute for Health Research Collaboration for Leadership in Applied Health Research and Care West (NIHR CLAHRC West) at University Hospitals Bristol NHS Foundation Trust, UK

Richard Morris, Population Health Sciences, Bristol Medical School, University of Bristol, Bristol, UK

Sarah Purdy, Population Health Sciences, Bristol Medical School, University of Bristol, Bristol, UK,

Word Count: 3862

For peer review only

Abstract

Introduction: Electronic palliative care coordination systems (EPaCCS) aim to support people approaching the end-of life to receive consistent care, according to their wishes, that is coordinated effectively across multiple care sectors. They are in use across the UK although empirical evidence into their effectiveness is poor. This paper presents a protocol of a mixed-methods study, to understand how, and by whom, EPaCCS are being used and whether EPaCCS are enabling Healthcare Professionals (HCPs) to coordinate patients' end-of-life (EOL) care.

Methods and Analysis: This is a mixed-methods study, carried out within a realist paradigm, to evaluate the impact of an EPaCCS on EOL care as provided by a Clinical Commissioning Group (CCG) in England. This study has two aims: 1. Describe the socio-demographic characteristics of patients who die with an EPaCCS record, their underlying cause of death and place of death and compare these with patients who die without an EPaCCS record. 2. Explore the impact of an EPaCCS on the experience of receiving EOL care for patients and their carers, and understand HCPs' views and experiences of utilising an EPaCCS to coordinate care for their patients. The study will be conducted in five phases: (1) development of the initial programme theory; (2) focus group with CCG stakeholder board; (3) individual interviews with HCPs, patients, current and bereaved carers; (4) retrospective cohort study of routinely collected data on EPaCCS usage and (5) data analysis and synthesis of study findings.

Ethics and dissemination: The study has been approved by NHS South West – Frenchay Research Ethics Committee (REC reference number: 18/SW/0198). Findings will be published in a wide range of outputs targeted at key audiences.

Strengths and limitations of this study

For peer review only

- Using a theory-driven realist evaluation approach, findings from this study are expected to generate contextually relevant evidence for other care coordination systems, as well as informing EPaCCS commissioning decision-making nationally.
- The study will test and refine these theories using a mixed methods approach, enhancing the credibility of the evaluation findings.
- This study addresses the need for qualitative research into the use of EPaCCS, offering much needed insight into patient and carers' experiences of EPaCCS.
- The study will investigate the impact of only one of the many EPaCCS developed and implemented in the UK.
- Some may perceive the small sample sizes in the qualitative sections of the study as a weakness. However, the aim of the study is not to find a robust causal mechanism for EPaCCS, but to unpack the contexts and mechanisms that work in certain circumstances. Following this we will highlight the factors which are crucial for effectiveness, providing essential guidance for implementation of EPaCCS in other localities.

Key words

Palliative Care, Quality in Health Care, Primary Care, Qualitative Research

For peer review only

Background

People at the end-of-life (taken here to mean in the last 12 months of life) frequently receive care from a wide variety of teams and organisations. Much of this care is accessed in the out-of-hours period (overnight and at the weekend), when they are unlikely to see a Healthcare Professional (HCP) who knows them well. Out-of-hours provision of palliative care (defined by NICE as "the active holistic care of patients with advanced, progressive illness" (1)) has been identified as a key priority for future research by the Palliative and End of Life Care Priority Setting Partnership, initiated in 2013 by Marie Curie (2). This process identified the top 10 unanswered research questions and the question with the highest priority was, "What are the best ways of providing palliative care outside of working hours to avoid crises and help patients to stay in their place of choice?"

Continuity of care is important for anyone with complex health and social care needs, but particularly for those at the end-of-life (EOL) (3). Until recently HCPs have communicated patients' end of life care plans, to other HCPs, by means of a variety of methods, including shared end of life care registers, letters, faxes and telephone and/or face to face conversations. Despite this, a lack of information sharing has been repeatedly cited as a barrier to the provision of good quality EOL care outside of normal working hours (4,5,6). Recent studies looking at the experiences and needs of people seeking palliative care out-of-hours found that most patients expect the HCP to be able to access a summary of their complex medical history and many voiced concerns that their full record could not be accessed by out-of-hours clinicians (5,7). For some patients, lack of access to their notes is a deterrent to accessing care out-of-hours (6).

Nationally, the policy drive to address the issue of information continuity has resulted in the development of Electronic Palliative Care Coordination Systems, or EPaCCS (5,8,9,10). These records are usually completed by the patients' GP or community nurse, including

For peer review only

patients' advance care wishes, and are accessible across multiple care sectors (10). The purpose of EPaCCS is to provide a shared local record for health and social care professionals, with key information about an individual approaching the EOL, including their expressed preferences for care. In accordance with the Quality Statements in the NICE End of Life Care Standard for Adults (11) the intent is for EPaCCS to support people approaching the EOL to receive consistent care that is coordinated effectively across all relevant settings.

An EPaCCS record can take various forms, including web-based electronic registers, systems based on sharing care summaries or care plans, alongside patients' electronic records. They store a dynamic record of a patient's medical condition, treatment, wishes and preferences, and provide information about the medication a patient is receiving, contact details of any carers, and services involved in providing care and support to the patient.

Sharing information about patients' EOL care has the potential to improve coordination and communication across care settings (12). It may reduce the chance of emergency department attendance, hospital admission and dying in hospital (8, 13).

It is now recognised that place of death is unlikely to be the most important factor in achieving a good death and a recent UK study has proposed that it is the presence of loved ones that is more important than the physical location (14). However, death in preferred place remains a significant measure of quality of death (15) and, according to the Voices survey of bereaved people, despite 81% of respondents believing that the deceased had wanted to die at home less than a quarter of people actually achieve this (16, 17).

Quantitative studies have shown striking differences in place of death with EPaCCS but are potentially biased and confounded (12). A more recent study challenges these assumptions, suggesting that the increase in home deaths could in fact be due to selection bias (few secondary care colleagues used the systems and therefore hospital deaths are not captured)

For peer review only

(10). The findings of this study also underscore the importance of qualitative approaches, which can offer crucial insights into what is happening on the ground, away from broad claims of EPaCCS benefits arrived at solely through quantitative methods. Without understanding the experiences of patients and carers, together with the perspectives of HCPs, it is difficult to evaluate the effectiveness of EPaCCS (18).

Technology in isolation is not guaranteed to bring benefit and the initiation of an EPaCCS relies on healthcare professionals initiating conversations about death and dying. There is evidence that these conversations are difficult for HCPs (19), with many choosing to avoid the conversation altogether (20). What impact EPaCCS has upon these conversations, if any, is not clear.

A recent independent evaluation of EPaCCS found that "it was not possible to demonstrate that EPaCCS was making any difference to the care patients were receiving at the end-of-life because the range of clients for whom EPaCCS was being used remains focused on cancer, and the ability of EPaCCS systems to report on progress and outcomes remains generally poor" (21).

We therefore do not know if EPaCCS acts to improve practice or whether it simply documents and reflects what is already taking place in practice (21). Indeed, the need to gather evidence of effectiveness of EPaCCS before widespread and uncritical adoption by the NHS is key.

To summarise, very little research has been carried out to understand how, and by whom, EPaCCS are being used and, perhaps more importantly, whether EPaCCS are enabling HCPs to support patients' EOL wishes. Rigorous evaluation and research are urgently needed to investigate to what extent EPaCCS influence services working together to support 'a good death', the outcome that stakeholders think is of most importance (22).

For peer review only

Aims, Objectives and Research Questions

The study has two aims. These are to:

1. Describe the socio-demographic characteristics of patients who die with an EPaCCS record, their underlying cause of death and place of death and compare these with patients who die without an EPaCCS record.

2. Explore the impact of an EPaCCS on the experience of receiving EOL care for patients and their carers, and understand HCPs' views and experiences of utilising an EPaCCS to provide palliative care to their patients.

Project methodology

Study setting

This study will be implemented in England, where one Clinical Commissioning Group (CCG) has recently developed and rolled out an EPaCCS. This involved the dissemination of an EMIS template, which was circulated to General Practices, to help ensure consistent data entry and coding. EMIS is the most widely used primary care clinical system in the UK and allows real-time patient information to be shared securely between different organisations. All practices in the CCG area are EMIS users and it is now also used by the local hospice and many of the community nursing teams.

The EPaCCS template was developed following extensive local clinical consultation and the National Information Standard (23). Although some organisations within the CCG area (ambulance service, secondary and social care) are non-EMIS users, information from the EPaCCS template can be viewed across the local health community, via the integrated digital care record used by health and social care professionals in the CCG area, which went live at the end of February 2018. The integrated digital care record contains some of the information

For peer review only - http://bmjopen.bmj.com/site/about/guidelines.xhtml

held at GP practices, hospital departments, community services, mental health trusts, out of hours services and local authorities across the CCG area, combining it into a single, shared digital record.

Conceptual framework

This study will draw on a realist evaluation approach (24). A randomised, experimental study design is not possible as the implementation of EPaCCS has been strongly advocated and promoted by NHS England, with 83% of Clinical Commissioning Groups (CCGs) in England reported to have an operational EPaCCS, or be in the planning stages, by 2013 (25). The CCG had recently operationalised an EPaCCS at the time of study design.

By their nature, EPaCCS involve multiple organisations and a multidisciplinary style of work. It therefore requires a novel methodological approach to evaluation as described in this protocol.

Realist evaluation is a theory-driven approach designed for evaluating complex interventions, such as EPaCCS, where the outcomes are influenced by the way the intervention is delivered and in what context. Due to this complexity, any evaluation of EPaCCS seeking to determine linear causal relationships or simply find out if the intervention "works" is unlikely to be useful. Pawson and Tilley (24), the developers of realistic evaluation, suggest that the results of an intervention (outcomes) are dependent on the introduction of appropriate reasoning and resources (mechanisms) and how these then interact with existing social and cultural condition (contexts). For the purposes of this study, we have defined context (C), mechanism (M) and outcome (O) in Figure 1.

Figure 1 – Definition of Context, Mechanism and Outcome

A realist approach acknowledges that complex interventions only ever work for certain people, in particular circumstances. The key task of a realist evaluation is to understand and

For peer review only

explain these patterns by asking the exploratory question: *what works, for whom and in what circumstances?*

According to Pawson, interventions or "programmes", such as the EPaCCS, are 'theories incarnate' and every programme has a theoretical underpinning (26). Before conducting a realist evaluation, the researchers must develop a theory, or theories, that explain what works, for whom, under what circumstances and how. This is sometimes known as the "initial programme theory" and is described through CMO conjectures. This theory, or theories, are then tested through the process of the evaluation.

The study will be conducted in five phases: (1) development of the initial programme theory; (2) focus group with CCG stakeholder board; (3) individual interviews with healthcare professionals (HCPs), patients, current and bereaved carers; (4) retrospective cohort study of routinely collected data on EPaCCS usage and (5) data analysis and synthesis of study findings.

Phase one: development of the initial programme theory (June-October 2018)

Phase one is complete and included identification of relevant literature from electronic searches of databases, such as Medline and Google Scholar. Reference lists of relevant papers were scanned, and citation searches conducted. Grey literature relating to policy and organisational-based material were sought by searching government and other specialist websites. The lead researcher's own experiences as a General Practitioner (GP) were used as "informed guesswork" (27) and initial meetings were held with key stakeholders, including patients at the local hospice and commissioners. These initial engagements were informal and were patient and public involvement activities (detailed further below). They did not constitute formal interviews requiring ethics clearance.

For peer review only

The proposed implementation of EPaCCs was broken down and analysed, to understand different elements of this process (Table 1, Column 1). These elements, highlighted as important through literature searches and initial stakeholder engagement, were analysed and detailed into initial CMO statements (Table 1, Column 2). An overview of these CMO statements were then illustrated through a process diagram as illustrated in Figure 2.

At an early stage of the programme theory development, the CMO conjectures were reviewed by, and discussed with, Dr Justin Jagosh, Director of the Centre for Advancement in Realist Evaluation and Synthesis.

The initial programme theory forms a set of hypotheses on what the mechanisms may be, what groups may benefit most or least and the contextual factors that might be important to its success or failure. These hypotheses will be interrogated and tested in phases 2-5 of the study.

Figure 2 - Visual representation of the initial programme theory at a macro level.

Phase two: focus group with CCG stakeholder board (October 2018)

The CCG EOL care board is a multi-disciplinary, multi-organisational system board, whose members are high-level stakeholder representatives from across the CCG, including representatives from community nursing teams, primary care, the ambulance service (which serves a wider geographical area than the CCG area), local hospices, care homes and secondary care. The purpose of the board is to review the current commissioning across all geographical provision, ensuring unified pathways for community, primary and secondary providers to provide consistency for all patients, carers and staff across the system.

Board members will be invited to take part in a focus group following their attendance at the monthly board meeting. Focus groups allow for social interaction, which can help to reveal

For peer review only

issues and subsequent points of view that may not be prompted or discovered through individual interviews. This approach will help the study team to gain as wide an understanding as possible of the process of commissioning the EPaCCS and help to refine the initial programme theory.

Participants will be consented to take part in the study prior to the focus group by either LF or LP who will be facilitating. A topic guide will be used to steer the focus group and will enable the research team to test and refine the initial programme theory prior to commencing the in-depth interviews (see table 1).

It is anticipated that the focus group will last approximately 45 minutes and that approximately 5-10 participants will take part in it. The focus group will be audio taped and transcribed verbatim.

Table 1: Programme theory for the EPaCCs study, comprising the 17 context, mechanism and outcome (CMO) statements that inform the programme theory, and the questions that will be used, in the focus group with the end-of-life Board, to investigate each CMO statement.

EPaCCs Process	CMOs	Focus group questions
**Commissioning**	1. If the strategy behind the EPaCCs is definable, deliverable and measureable, the aim, purpose and outcomes of EPaCCS will be clear. ***(strategy)***	• How will you evaluate EPaCCS success?<br>• What are the markers of success for you?<br>• What is the CCGs long term vision for the EPaCCS?
	2. If HCPs engage with the EPaCCS positively on early usage and see it as an improvement on any previous EOL register, HCPs will engage positively with EPaCCS. ***(engagement)***	• Given that the previous EOL register was generally not well thought of, or used, how did the CCG plan to get HCPS on board?<br>• How do you think the EPaCCS has been received?
**Commissioning/Uptake/Adoption**	3. If the EPaCCS is well-publicised and marketed to all stakeholders HCPs will be aware of EPaCCS, understand the aim and purpose of the EPaCCS, and will initiate an EPaCCs template and/or access an EPaCCS record. ***(publicity)***	• How was the EPaCCS publicised and marketed to different groups of healthcare professionals (HCPS)? What are your views on how effective this has been?<br>• How aware do you think HCPs are of EPaCCS and do you think they understand its purpose and importance?
	4. If HCPs receive sufficient support and training, so that they know how to use it, they and will initiate an EPaCCs template and/or access an EPaCCS record. ***(training)***	• Can you tell us about the CCG strategy for providing training and support to different groups of HCPs in the EPaCCS rollout?<br>• What do you think about this, and how effective it has been?
**Uptake/Adoption**	5. If HCPs have the time and/or resources to learn a new system, an EPaCCS template will be initiated. ***(time & resources)***	• There are a significant number of GP practices that have not initiated an EPaCCS – do you have any thoughts about why this might be?

		• Do you think all HCPs will have the time and resources (i.e. they are connected to a computer, have internet and NHS network access) to learn and new system and access EPaCCS?
	6. If HCPs are incentivised to use EPaCCS, an EPaCCS template will be initiated. ***(incentives)***	• Do HCPs have other ways of obtaining the information contained on EPaCCs?<br>• What might these be, and are these ways better or worse, more reliable or less reliable?
**Uptake**	7. If the patient consents to information-sharing and storage of information about their care preferences, an EPaCCS template will be initiated. ***(information sharing)***	• For the EPaCCS to be effective, patients must consent to information-sharing, and the storage of information.<br>• Did you anticipate that this would raise any issues?
	8. If HCPs are near to a computer, are connected to the internet and have access to the GP EMIS record, an EPaCCS template will be initiated. ***(access to system)***	• There is a theory that because EPaCCS is an electronic record, presently only updateable by the GP on EMIS, that this will have an impact on the ability of others to access it and update it and own it. Do you see this as an issue?<br>• What impact do you think this might present?
	9. If HCPs feel able/comfortable having advanced care planning (ACP) conversations with patients, an EPaCCS template will be initiated. ***(ACP conversations)***	• How do you think HCPs feel about having ACP conversations with patients?<br>• Research suggests that patients with non-malignant diagnoses are less likely to be added to EPaCCS.<br>• Do you think this is the case and if so why? Are there other patient groups who might be underrepresented on the EPaCCS?
	10. If HCPs feel that the EPaCCS facilitates, potentially difficult, ACP conversations an EPaCCS template will be initiated. ***(ACP conversations)***	• Some would argue that the EPaCCS template might facilitate ACP conversations with patients – what are your thoughts on this?
	11. If the patient is willing, and has capacity to have ACP conversations, an EPaCCS template will be initiated. ***(ACP conversations)***	• Patients can only record their wishes if they are able to have a conversation with an HCP – what issues do you think this might present?
**Adoption**	12. If EOLC information about a patient can be accessed more efficiently in other ways (i.e. speaking with carer or reading other sources of information) the information on the EPaCCS template may not be accessed. ***(single point of access)***	• Are there any other sources of information that HCPs might access to establish the EOL wishes and needs of a patient and do you think they present an issue of the uptake of EPaCCS?
	13. If HCPs are near to a computer, are connected to the internet and have access to the NHS Network an EPaCCs template will be accessed. ***(access to system)***	• There is a theory that because EPaCCS is an electronic record, presently only updateable by the GP on EMIS, that this will have an impact on the ability of others to access it and update it and own it.<br>• Do you see this as an issue? What impact do you think this might present?
**Adoption/Care Coordination**	14. If the information does not reflect the current wishes of the patient, care may not be aligned with the patients' preferences. ***(patient preferences)***	• Do you feel that the EPaCCs adequately reflects patient's wishes and preferences for care?
	15. If the patient does not have clear or clinically meetable preferences, or their wishes are subject to frequent change, care may not be aligned with the patient's wishes. ***(patient preferences)***	• Do you feel the EPaCCS adequately reflects the patient's/carer's wishes and preferences regarding end of life care and do you feel these wishes are deliverable? If not, why might this be and what needs to be improved?
**Care Coordination**	16. If HCPs access EPaCCS and consider the information contained within it to be trustworthy (current, relevant, detailed and useful) care will be coordinated by EPaCCs and this care will align with the patient's	• Do you think the EPaCCS contains all the information HCPs need to enact their patient's wishes and coordinate their patient's care?<br>• Do you consider it to be current, relevant, detailed and useful? If not, why might this be and what needs to be improved?

	wishes. ***(trustworthiness of EPaCCS)***	
	17. If EPaCCS does not enhance or improve the care that is already being delivered care may not be coordinated by EPaCCS, consistent or reflect the patients' preferences. ***(patient outcomes)***	• What are your thoughts on the notion that: 'The EPaCCS is not coordinating care, it is simply recording what is already being done'

Phase three: individual interviews with healthcare professionals (HCPs), patients, current and bereaved carers (November 2018 – July 2019)

Healthcare professionals (HCPs)

HCPs from community nursing teams, primary care, the ambulance service, the local hospices, care homes and secondary care will be invited to participate.

GPs working in practices within the CCG area will be invited to support the study and the study team will purposively sample from a list of practices, to include practices that are high-users of EPaCCS and low users of EPaCCS based on data compiled by the CCG.

The research team will purposively sample HCPs who express an interest in participating according to gender, age, and profession to ensure maximum variation in the sample. All interviews with HCPs will take place over the telephone for both pragmatic and methodological reasons. Conducting interviews over the telephone will reduce the time and cost to the study that may be involved in travelling to interviews and well-planned telephone interviews can gather the same material as those held face to face (28).

A topic guide, informed by the evolving programme theory, will be used to ensure consistency across the interviews. This will enable the research team to compare the views of each group at the stage of data analysis. Interviews will last approximately 30 minutes and it is anticipated that approximately 3-5 HCPs will be interviewed from each group (18-30 in total).

Patients, current and bereaved carers

For peer review only

Patients will be approached to take part in interviews through their GP surgeries or the local hospice. The research team will purposively sample from a list of practices, to include practices that are high-users of EPaCCS and low users of EPaCCS based on data compiled by the CCG which makes clear the number of EPaCCS templates initiated according to practice. Selected practices will be asked to identify patients, aged 18 and over, receiving EOL care, who they consider might be eligible to take part in the study.

Inclusion criteria:

1. Capacity to give informed consent
2. Aged 18 and over
3. Prognosis 12 months or less as identified by their GP (patient aware of this prognosis)

Potential participants will be sampled purposively to include patients across the age range, with and without an EPaCCS record, with both malignant and non-malignant health conditions.

To recruit patients to the study from the local hospice we will liaise with key clinical staff, who will be responsible for identifying appropriate patients. Once again, potential participants will be sampled purposively to include patients across the age range, with and without an EPaCCS record, with both malignant and non-malignant health conditions.

Alongside their own study information pack, all patients will be given a carer information pack which they can choose to give to their carer if they are happy for their carer to participate in the study.

GPs will also be asked to identify recently bereaved carers (between 8 weeks and 6 months of the death of their relative), who they consider might be eligible to take part in the study. GPs will be sent details of how to perform an appropriate search within EMIS to identify potential participants.

For peer review only

Interviews with carers will be conducted one-to-one with the interviewer. Interviews with patients will be conducted either one-to-one with the interviewer or in the presence of their carer, according to the wishes of the patient.

Interviews will last approximately 45 minutes and it is anticipated that approximately 15 patients will be interviewed (10 EPaCCS patients and 5 non-EPaCCS patients) and 10 carers (to include both current and bereaved). All interviews will be audio taped and transcribed verbatim.

Phase four: retrospective cohort study of routinely collected data on EPaCCS usage (March 2019 – July 2019)

EMIS data will be accessed to identify all patients, aged 18 and over, who die in the CCG area between 22nd February 2018 and 21st February 2019. Agreements are in place with the CCG to obtain this data. Patients will be identified as either having an EPaCCS record (EPaCCS patient) or not (non-EPaCCS patient), using EMIS coding.

EMIS will be used to characterise both EPaCCS and non-EPaCCS patients in terms of their gender, ethnicity and postcode (as a proxy for socio-economic status according to their Index of Multiple Deprivation Score). Data will be extracted to describe:

1. The proportion of patients that die with an EPaCCS record.
2. When the EPaCCS record is initiated (i.e. how many months/days prior to the patient's death), and by whom.
3. How frequently the EPaCCS record is updated once opened, and who makes any changes.
4. The underlying cause and place of death for EPaCCS and non-EPaCCS patients.

For peer review only

5. The number of hospital admissions and Emergency Department attendances for EPaCCS and non-EPaCCS patients in the last 12 months of life.

Descriptive data will be collected, by the CCG, on EPaCCS usage, including the number of records created by each GP surgery in the CCG area. Data from the integrated digital care record will be accessed to describe which HCPs (GPs, community nurses, hospice HCPs, ambulance HCPs and secondary care clinicians) are accessing these shared end-of-life care records.

Phase five: data analysis and synthesis of study findings (October 2018 – October 2019)

Quantitative methods

Quantitative data will be analysed using Stata v15 and reported using descriptive statistics. Within the context of this realist evaluation, we were keen to use the quantitative data to address a single hypothesis, namely whether nominal possession of an EPaCCS record was associated with increased chance of dying at home. However, logistic regression will be used to determine the adjusted odds ratio and 95% confidence intervals for the associations between having an EPaCCS record and dying at home, considering other factors of interest, including, but not limited to: age, sex, deprivation and underlying cause of death.

Of approximately 8,000 deaths occurring in the CCG area over the year of study, we expect around 10% (800 deaths) of patients to have an EPaCCS (10). If the proportion of deaths occurring at home is expected to be 25% among those without an EPaCCS, we would have over 99% power to detect an absolute increase of 10% to 35% among those with an EPaCCS. The power would be about 84% if the proportion were increased by 5% to 30%.

Descriptive statistics will be employed to report EPaCCS usage.

Qualitative methods

For peer review only

Data analysis will be conducted using a realist approach informed by Jackson and Kolla's realistic evaluation analysis method (29). This analytic process will involve the following steps:

1. Coding individual units (a discrete C, M or O) within the narratives of the interviews.
2. Identifying the complex connections that link these codes together into dyads or triads.
3. Subsuming the linked codes into themes using thematic analysis (29).

Analysis will begin shortly after data collection starts and be ongoing and iterative. Analysis will inform further data collection: for instance, analytic insights from data gathered in earlier interviews will be used to develop and adapt the programme theory and in turn, identify any changes that need to be made to the topic guide for use during later interviews. The study will generate new programme theories to explain how the EPaCCS works, for whom, and any contextual influences and constraining factors that affect their initiation and usage. Emerging analysis and findings will be discussed with PPI representatives, to explore and clarify findings.

Qualitative and quantitative data will be collected concurrently, giving equal weight to each (30). Data will be triangulated in order to test and refine the programme theories, accepting that any findings are fallible and with time and further study new data are bound to emerge (31). The synthesised study findings will establish the potential outcomes of EPaCCS, identify the underlying mechanisms which explain how they produce these effects and highlight the key contextual factors that affect their success or failure. Recommendations can then be made for the development and implementation of EPaCCS.

Patient and Public Involvement

For peer review only

To support the development of this study protocol, members of the study team (LF and LP) presented and discussed an outline proposal of this study to patients, staff and carers at the local hospice on two separate occasions in April 2018. Approximately 10 participants voluntarily took part in these, semi-structured, discussions, in which we asked specific questions concerning ethical and methodological issues. Participants were also encouraged to ask any questions. These meetings raised several important issues which have been incorporated into the design of this study. Such issues included allowing patients the choice of whether to have a carer sit alongside them during their interview and which HCPs they felt it was important that the study team spoke to, due to the involvement they had in providing care for patients. The meetings also discussed what terms, wording and questions would be acceptable to patients and carers to read and hear in the study information documents and interviews.

At the end of both meetings, patients and carers were invited to continue to support the design of the study should they wished. Two members came forward expressing a wish to be more actively involved in the study. They have kindly been involved in reviewing all the study literature, including the topic guides, study information sheets and the lay summary for this protocol. It is hoped that they will wish to continue their involvement with the study. This will include informing the content of materials for lay audiences, drawing links to groups and forums that the research team may be unaware of, and supporting the study team with the interpretation and dissemination of study findings. To ensure ongoing PPI, all patients and carers taking part in the study will be invited to support the ongoing development of the study.

Ethics and dissemination

For peer review only

The study has been approved by NHS South West – Frenchay Research Ethics Committee (REC reference number: 18/SW/0198).

The research team will disseminate the findings to a range of stakeholders. We will draw on the networks and expertise of the local CCG end-of-life care board to disseminate the research outputs widely and appropriately. Key audiences include patient and carer organisations, GPs and community nursing teams in primary care, ambulatory services and care home staff, HCPs working in secondary palliative care services and hospices, managers and directors within healthcare organisations with responsibility to provide high-quality services within budget and healthcare policymakers, nationally and internationally.

Acknowledgements

We would like to thank all the patients, carers and HCPs who helped to support the PPI in development of this study.

We would also like to thank Dr Justin Jagosh, Director of the Centre for Advancement in Realist Evaluation and Synthesis (https://realistmethodology-cares.org/), for his support and guidance in developing the initial programme theory.

Contributors

LP was the principal investigator for this qualitative study. LF, MF, RM and SP all contributed to the development of the study protocol. LP and LF drafted this manuscript. All authors contributed to the editing of the final manuscript and the refining of its intellectual content.

Funding

For peer review only

LP's time is paid for by a NIHR School for Primary Care Research GP Career Progression Fellowship. This fellowship funding will also meet the research costs. LF's time is paid for by NIHR Research Capability Funding. MF's time is supported by the NIHR Collaboration for Leadership in Applied Health Research and Care West (NIHR CLAHRC West). The study is supported by the NIHR West of England Clinical Research Network. The views expressed in this publication are those of the authors and not necessarily those of the NHS, the National Institute for Health Research or the Department of Health and Social Care.

Competing interests

We have read and understood BMJ policy on declaration of interests and declare that we have no competing interests.

References

1. National Institute for Health and Care Excellence (2004). Improving supportive and palliative care for adults with cancer. Cancer service guideline [CSG4].
2. Palliative and end of life care Priority Setting Partnership: Putting patients, carers and clinicians at the heart of palliative and end of life care research. January 2015. Available at: https://www.mariecurie.org.uk/globalassets/media/documents/research/PeolcPSP_Final_Report.pdf (accessed 30/05/18)
3. Barker I, Steventon A, Deeny SR. Association between continuity of care in general practice and hospital admissions for ambulatory care sensitive conditions: cross sectional study of routinely collected, person level data. *BMJ* 2017;356:j84

For peer review only

4. Schweitzer B, Blankenstein N, Deliens L, Van Der Horst H. Out-of-hours palliative care provided by GP co-operatives in the Netherlands: A focus group study. *European Journal of General Practice*, 2011; 17: 160–166.

5. Hall S, Murchie P, Campbell C and Murray SA. Introducing an electronic Palliative Care Summary (ePCS) in Scotland: patient, carer and professional perspectives. *Family Practice* 2012; 29:576–585.

6. Richards SH, Winder R, Seamark C, Seamark D, Avery S. Gilbert J, Barwick A and Campbell JL. 2011: The experiences and needs of people seeking palliative health care out-of-hours: a qualitative study. *Primary Health Care Research & Development* 12(2), 165–78; available from: PM:21457601.

7. Leydon GM, Shergill NK, Campion-Smith C, et al. Discontinuity of care at end of life: a qualitative exploration of OOH end of life care. *BMJ Supportive & Palliative Care* 2013;3:412-421.

8. Purdy S, Lasseter G, Griffin T, Wye L. Impact of the Marie Curie Cancer Care Delivering Choice Programme in Somerset and North Somerset on place of death and hospital usage: a retrospective cohort study. *BMJ Supportive & Palliative Care* 2014;0:1–6.

9. Asprey A, Richards SH, Wright C, Seamark C, Seamark D and Moxon J. Transferring information to an out-of-hours primary care service for patients with palliative care needs: an action research study to improve the use of handover forms. *Primary Health Care Research & Development* 2013; 14: 7–20.

10. Wye L, Lasseter G, Simmonds B, Duncan L, Percival J and Purdy S. Electronic palliative care coordinating systems (EPaCCS) may not facilitate home deaths: A mixed methods evaluation of end of life care in two English counties. *Journal of Research in Nursing*, 2016, Vol. 21(2) 96–107

For peer review only

11. National Institute for Health and Clinical Excellence (2011) End of Life Care for Adults. NICE Quality Standard [QS13]

12. Petrova M, Riley J, Abel J and Barclay S. Crash course in EPaCCS (Electronic Palliative Care Coordination Systems): 8 years of successes and failures in patient data sharing to learn from. *BMJ Supportive & Palliative Care* 2016;0:1–9.

13. Ali AA, Adam R, Taylor D, et al. Use of a structured palliative care summary in patients with established cancer is associated with reduced hospital admissions by out-of-hours general practitioners in Grampian. BMJ Support Palliat Care2013;3:452–5.doi:10.1136/bmjspcare-2012-000371

14. Turner V, Flemming K. Socioeconomic factors affecting access to preferred place of death: A qualitative evidence synthesis. *Palliative medicine* 2019 Mar 8:0269216319835146.

15. Department of Health (2016) Our Commitment to you for end of life care: the Government Response to the Review of Choice in End of Life Care. Available at: https://assets.publishing.service.gov.uk/government/uploads/system/uploads/attachment_data/file/536326/choice-response.pdf (accessed 21/03/19)

16. Office for National Statistics. National Survey of Bereaved People (VOICES): England, 2015. Available at: https://www.ons.gov.uk/peoplepopulationandcommunity/healthandsocialcare/healthcaresystem/bulletins/nationalsurveyofbereavedpeoplevoices/england2015#preferences-and-choice-at-the-end-of-life (accessed 30/05/18)

17. Public Health England, End of Life Care Profiles. Available at: https://fingertips.phe.org.uk/profile/end-of-life (accessed 30/05/18)

18. Allsop MJ, Kite S, McDermott S, Penn N, Millares-Martin P and Bennett MI. Electronic palliative care coordination systems: Devising and testing a methodology for evaluating documentation, *Palliative Medicine* 2017 May; 31(5): 475–482.

19. Fallowfield L and Jenkins V. Communicating sad, bad, and difficult news in medicine. *The Lancet*, 2004;*363*(9405), 312-319.

20. Gibbins J, McCoubrie R, and Forbes K. Why are newly qualified doctors unprepared to care for patients at the end of life? *Medical education*;2011, *45*(4), 389-399.

21. Whole Systems Partnership. (2016) *Independent evaluation of Electronic Palliative Care Co-ordination Systems (EPaCCS) in England: Final Report*, March 2016

22. Wye L, Lasseter GM, Percival JF, Simmonds BAJ, Duncan LJ, Purdy S. Independent Evaluation of the Marie Curie Cancer Care Delivering Choice Programme in Somerset and North Somerset. Centre for Primary Health Care, Department of Social and Community Medicine, The University of Bristol, 2012.

23. NHS Digital (2015). SCCI1580: Palliative Care Co-ordination: Core Content. Available at: https://digital.nhs.uk/data-and-information/information-standards/information-standards-and-data-collections-including-extractions/publications-and-notifications/standards-and-collections/scci1580-palliative-care-co-ordination-core-content (accessed 23/01/19)

24. Pawson R and Tilley N. Realistic Evaluation. London: Sage, 2005.

25. National End of Life Care Intelligence Network. (2014). *Electronic Palliative Care Co-ordination Systems (EPaCCS) in England: Survey of clinical commissioning groups (2013).*

26. Pawson R. Evidence based policy: A realist perspective. London: Sage, 2006.

For peer review only

27. Greenhalgh T, Wong G, Jagosh J, et al Protocol—the RAMESES II study: developing guidance and reporting standards for realist evaluation BMJ Open 2015;5:e008567. doi: 10.1136/bmjopen-2015-008567

28. Sturges JE, Hanrahan KJ. Comparing telephone and face-to-face qualitative interviewing: a research note. Qual Res. 2004 Apr;4(1):107-18.

29. Jackson SF, Kolla G. A new realistic evaluation analysis method: linked coding of context, mechanism, and outcome relationships. American Journal of Evaluation. 2012 Sep;33(3):339-49.

30. Hanson WE, Creswell JW, Clark VL, Petska KS, Creswell JD. Mixed methods research designs in counseling psychology. Journal of counseling psychology. 2005 Apr;52(2):224.

31. Emmel N, Greenhalgh J, Manzano A, Monaghan M, Dalkin S (Eds). Doing realist research. London: Sage, 2018.

For peer review only

Figure 1 – Definition of Context, Mechanism and Outcome

Context

Something that existed prior to the introduction of the EPaCCS, for example cultural views and beliefs around talking about death and dying and Advanced Care Planning (ACP) conversations. Contexts might also refer to the setting, (e.g. primary or secondary care) or patient characteristics (e.g. underlying diagnosis, socio-demographics, mental capacity).

Mechanism

The intended or unintended resources created by an intervention and the response to those resources (cognitive, emotional, motivational etc) by Healthcare Professionals (HCPs), patients and carers. Mechanisms can pertain to why HCPs and patients choose (or choose not) to utilise the EPaCCS.

Outcome

An outcome will define the result of the EPaCCS whether intended (did the project succeed against the criteria it set itself at the outset), and also the unplanned and/or unexpected impacts.

Informed by (24) and (26).

For peer review only

Figure 2 – Visual representation of the initial programme theory at a macro level. See table 1 for associated CMO statements.

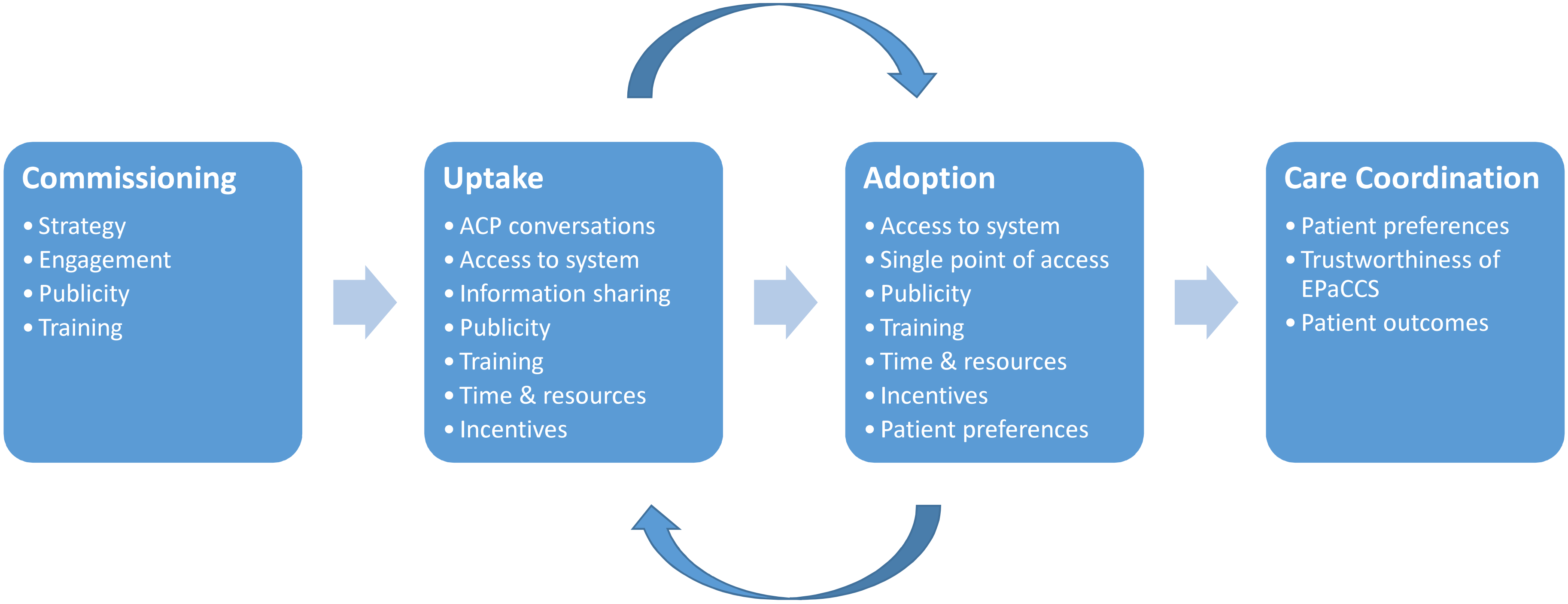

BMJ Open

THE IMPACT OF ELECTRONIC PALLIATIVE CARE COORDINATION SYSTEMS (EPaCCS) ON CARE AT THE END OF LIFE ACROSS MULTIPLE CARE SECTORS, IN ONE CLINICAL COMMISSIONING GROUP AREA, IN ENGLAND: A REALIST EVALUATION PROTOCOL

Journal:	*BMJ Open*
Manuscript ID	bmjopen-2019-031153.R2
Article Type:	Protocol
Date Submitted by the Author:	12-Nov-2019
Complete List of Authors:	Pocock, Lucy; University of Bristol, Centre for Academic Primary Care<br>French, Lydia; University of Bristol, Centre for Academic Primary Care<br>Farr, Michelle; University of Bristol, NIHR CLAHRC West, Bristol Medical School<br>Morris, Richard; University of Bristol, School of Social & Community Medicine<br>Purdy, Sarah; University of Bristol, Centre for Academic Primary Care
**Primary Subject Heading**:	Palliative care
Secondary Subject Heading:	Health services research
Keywords:	PALLIATIVE CARE, Quality in health care < HEALTH SERVICES ADMINISTRATION & MANAGEMENT, PRIMARY CARE, QUALITATIVE RESEARCH

SCHOLARONE™ Manuscripts

I, the Submitting Author has the right to grant and does grant on behalf of all authors of the Work (as defined in the below author licence), an exclusive licence and/or a non-exclusive licence for contributions from authors who are: i) UK Crown employees; ii) where BMJ has agreed a CC-BY licence shall apply, and/or iii) in accordance with the terms applicable for US Federal Government officers or employees acting as part of their official duties; on a worldwide, perpetual, irrevocable, royalty-free basis to BMJ Publishing Group Ltd ("BMJ") its licensees and where the relevant Journal is co-owned by BMJ to the co-owners of the Journal, to publish the Work in this journal and any other BMJ products and to exploit all rights, as set out in our licence.

The Submitting Author accepts and understands that any supply made under these terms is made by BMJ to the Submitting Author unless you are acting as an employee on behalf of your employer or a postgraduate student of an affiliated institution which is paying any applicable article publishing charge ("APC") for Open Access articles. Where the Submitting Author wishes to make the Work available on an Open Access basis (and intends to pay the relevant APC), the terms of reuse of such Open Access shall be governed by a Creative Commons licence – details of these licences and which Creative Commons licence will apply to this Work are set out in our licence referred to above.

Other than as permitted in any relevant BMJ Author's Self Archiving Policies, I confirm this Work has not been accepted for publication elsewhere, is not being considered for publication elsewhere and does not duplicate material already published. I confirm all authors consent to publication of this Work and authorise the granting of this licence.

For peer review only

THE IMPACT OF ELECTRONIC PALLIATIVE CARE COORDINATION SYSTEMS (EPaCCS) ON CARE AT THE END OF LIFE ACROSS MULTIPLE CARE SECTORS, IN ONE CLINICAL COMMISSIONING GROUP AREA, IN ENGLAND: A REALIST EVALUATION PROTOCOL

Corresponding author

Lucy V Pocock, Population Health Sciences, Bristol Medical School, University of Bristol, Canynge Hall, 39 Whatley Road, Bristol, BS8 2PS

Lucy.pocock@bristol.ac.uk, Tel: 07810 002320

Co-authors

Lydia R M French, Population Health Sciences, Bristol Medical School, University of Bristol, Bristol, UK

Michelle Farr, The National Institute for Health Research Collaboration for Leadership in Applied Health Research and Care West (NIHR CLAHRC West) at University Hospitals Bristol NHS Foundation Trust, UK

Richard Morris, Population Health Sciences, Bristol Medical School, University of Bristol, Bristol, UK

Sarah Purdy, Population Health Sciences, Bristol Medical School, University of Bristol, Bristol, UK,

Word Count: 3862

For peer review only

Abstract

Introduction: Electronic palliative care coordination systems (EPaCCS) aim to support people approaching the end-of life to receive consistent care, according to their wishes, that is coordinated effectively across multiple care sectors. They are in use across the UK although empirical evidence into their effectiveness is poor. This paper presents a protocol of a mixed-methods study, to understand how, and by whom, EPaCCS are being used and whether EPaCCS are enabling Healthcare Professionals (HCPs) to coordinate patients' end-of-life (EOL) care.

Methods and Analysis: This is a mixed-methods study, carried out within a realist paradigm, to evaluate the impact of an EPaCCS on EOL care as provided by a Clinical Commissioning Group (CCG) in England. This study has two aims: 1. Describe the socio-demographic characteristics of patients who die with an EPaCCS record, their underlying cause of death and place of death and compare these with patients who die without an EPaCCS record. 2. Explore the impact of an EPaCCS on the experience of receiving EOL care for patients and their carers, and understand HCPs' views and experiences of utilising an EPaCCS to coordinate care for their patients. The study will be conducted in five phases: (1) development of the initial programme theory; (2) focus group with CCG stakeholder board; (3) individual interviews with HCPs, patients, current and bereaved carers; (4) retrospective cohort study of routinely collected data on EPaCCS usage and (5) data analysis and synthesis of study findings.

Ethics and dissemination: The study has been approved by NHS South West – Frenchay Research Ethics Committee (REC reference number: 18/SW/0198). Findings will be published in a wide range of outputs targeted at key audiences.

Strengths and limitations of this study

For peer review only

- Using a theory-driven realist evaluation approach, findings from this study are expected to generate contextually relevant evidence for other care coordination systems, as well as informing EPaCCS commissioning decision-making nationally.
- The study will test and refine these theories using a mixed methods approach, enhancing the credibility of the evaluation findings.
- This study addresses the need for qualitative research into the use of EPaCCS, offering much needed insight into patient and carers' experiences of EPaCCS.
- The study will investigate the impact of only one of the many EPaCCS developed and implemented in the UK.
- The qualitative component has a potentially small sample size, however, the aim of the study is not to find a robust causal mechanism for EPaCCS, but to unpack the contexts and mechanisms that work in certain circumstances.

Key words

Palliative Care, Quality in Health Care, Primary Care, Qualitative Research

For peer review only

Background

People at the end-of-life (taken here to mean in the last 12 months of life) frequently receive care from a wide variety of teams and organisations. Much of this care is accessed in the out-of-hours period (overnight and at the weekend), when they are unlikely to see a Healthcare Professional (HCP) who knows them well. Out-of-hours provision of palliative care (defined by NICE as "the active holistic care of patients with advanced, progressive illness" (1)) has been identified as a key priority for future research by the Palliative and End of Life Care Priority Setting Partnership, initiated in 2013 by Marie Curie (2). This process identified the top 10 unanswered research questions and the question with the highest priority was, "What are the best ways of providing palliative care outside of working hours to avoid crises and help patients to stay in their place of choice?"

Continuity of care is important for anyone with complex health and social care needs, but particularly for those at the end-of-life (EOL) (3). Until recently HCPs have communicated patients' end of life care plans, to other HCPs, by means of a variety of methods, including shared end of life care registers, letters, faxes and telephone and/or face to face conversations. Despite this, a lack of information sharing has been repeatedly cited as a barrier to the provision of good quality EOL care outside of normal working hours (4,5,6). Recent studies looking at the experiences and needs of people seeking palliative care out-of-hours found that most patients expect the HCP to be able to access a summary of their complex medical history and many voiced concerns that their full record could not be accessed by out-of-hours clinicians (5,7). For some patients, lack of access to their notes is a deterrent to accessing care out-of-hours (6).

Nationally, the policy drive to address the issue of information continuity has resulted in the development of Electronic Palliative Care Coordination Systems, or EPaCCS (5,8,9,10). These records are usually completed by the patients' GP or community nurse, including

For peer review only

patients' advance care wishes, and are accessible across multiple care sectors (10). The purpose of EPaCCS is to provide a shared local record for health and social care professionals, with key information about an individual approaching the EOL, including their expressed preferences for care. In accordance with the Quality Statements in the NICE End of Life Care Standard for Adults (11) the intent is for EPaCCS to support people approaching the EOL to receive consistent care that is coordinated effectively across all relevant settings.

An EPaCCS record can take various forms, including web-based electronic registers, systems based on sharing care summaries or care plans, alongside patients' electronic records. They store a dynamic record of a patient's medical condition, treatment, wishes and preferences, and provide information about the medication a patient is receiving, contact details of any carers, and services involved in providing care and support to the patient.

Sharing information about patients' EOL care has the potential to improve coordination and communication across care settings (12). It may reduce the chance of emergency department attendance, hospital admission and dying in hospital (8, 13).

It is now recognised that place of death is unlikely to be the most important factor in achieving a good death and a recent UK study has proposed that it is the presence of loved ones that is more important than the physical location (14). However, death in preferred place remains a significant measure of quality of death (15) and, according to the Voices survey of bereaved people, despite 81% of respondents believing that the deceased had wanted to die at home less than a quarter of people actually achieve this (16, 17).

Quantitative studies have shown striking differences in place of death with EPaCCS but are potentially biased and confounded (12). A more recent study challenges these assumptions, suggesting that the increase in home deaths could in fact be due to selection bias (few secondary care colleagues used the systems and therefore hospital deaths are not captured)

For peer review only

(10). The findings of this study also underscore the importance of qualitative approaches, which can offer crucial insights into what is happening on the ground, away from broad claims of EPaCCS benefits arrived at solely through quantitative methods. Without understanding the experiences of patients and carers, together with the perspectives of HCPs, it is difficult to evaluate the effectiveness of EPaCCS (18).

Technology in isolation is not guaranteed to bring benefit and the initiation of an EPaCCS relies on healthcare professionals initiating conversations about death and dying. There is evidence that these conversations are difficult for HCPs (19), with many choosing to avoid the conversation altogether (20). What impact EPaCCS has upon these conversations, if any, is not clear.

A recent independent evaluation of EPaCCS found that “it was not possible to demonstrate that EPaCCS was making any difference to the care patients were receiving at the end-of-life because the range of clients for whom EPaCCS was being used remains focused on cancer, and the ability of EPaCCS systems to report on progress and outcomes remains generally poor” (21).

We therefore do not know if EPaCCS acts to improve practice or whether it simply documents and reflects what is already taking place in practice (21). Indeed, the need to gather evidence of effectiveness of EPaCCS is vitally important, as it has already been widely and uncritically adopted by the NHS.

To summarise, very little research has been carried out to understand how, and by whom, EPaCCS are being used and, perhaps more importantly, whether EPaCCS are enabling HCPs to support patients’ EOL wishes. Rigorous evaluation and research are urgently needed to investigate to what extent EPaCCS influence services working together to support ‘a good death’, the outcome that stakeholders think is of most importance (22).

For peer review only

Aims, Objectives and Research Questions

The study has two aims. These are to:

1. Describe the socio-demographic characteristics of patients who die with an EPaCCS record, their underlying cause of death and place of death and compare these with patients who die without an EPaCCS record.

2. Explore the impact of an EPaCCS on the experience of receiving EOL care for patients and their carers, and understand HCPs' views and experiences of utilising an EPaCCS to provide palliative care to their patients.

Project methodology

Study setting

This study will be implemented in England, where one Clinical Commissioning Group (CCG) has recently developed and rolled out an EPaCCS. This involved the dissemination of an EMIS template, which was circulated to General Practices, to help ensure consistent data entry and coding. EMIS is the most widely used primary care clinical system in the UK and allows real-time patient information to be shared securely between different organisations. All practices in the CCG area are EMIS users and it is now also used by the local hospice and many of the community nursing teams.

The EPaCCS template was developed following extensive local clinical consultation and the National Information Standard (23). Although some organisations within the CCG area (ambulance service, secondary and social care) are non-EMIS users, information from the EPaCCS template can be viewed across the local health community, via the integrated digital care record used by health and social care professionals in the CCG area, which went live at the end of February 2018. The integrated digital care record contains some of the information

For peer review only

held at GP practices, hospital departments, community services, mental health trusts, out of hours services and local authorities across the CCG area, combining it into a single, shared digital record.

Conceptual framework

This study will draw on a realist evaluation approach (24). A randomised, experimental study design is not possible as the implementation of EPaCCS has been strongly advocated and promoted by NHS England, with 83% of Clinical Commissioning Groups (CCGs) in England reported to have an operational EPaCCS, or be in the planning stages, by 2013 (25). The CCG had recently operationalised an EPaCCS at the time of study design.

By their nature, EPaCCS involve multiple organisations and a multidisciplinary style of work. It therefore requires a novel methodological approach to evaluation as described in this protocol.

Realist evaluation is a theory-driven approach designed for evaluating complex interventions, such as EPaCCS, where the outcomes are influenced by the way the intervention is delivered and in what context. Due to this complexity, any evaluation of EPaCCS seeking to determine linear causal relationships or simply find out if the intervention "works" is unlikely to be useful. Pawson and Tilley (24), the developers of realistic evaluation, suggest that the results of an intervention (outcomes) are dependent on the introduction of appropriate reasoning and resources (mechanisms) and how these then interact with existing social and cultural condition (contexts). For the purposes of this study, we have defined context (C), mechanism (M) and outcome (O) in Figure 1.

Figure 1 – Definition of Context, Mechanism and Outcome

A realist approach acknowledges that complex interventions only ever work for certain people, in particular circumstances. The key task of a realist evaluation is to understand and

For peer review only

explain these patterns by asking the exploratory question: *what works, for whom and in what circumstances?*

According to Pawson, interventions or "programmes", such as the EPaCCS, are 'theories incarnate' and every programme has a theoretical underpinning (26). Before conducting a realist evaluation, the researchers must develop a theory, or theories, that explain what works, for whom, under what circumstances and how. This is sometimes known as the "initial programme theory" and is described through CMO conjectures. This theory, or theories, are then tested through the process of the evaluation.

The study will be conducted in five phases: (1) development of the initial programme theory; (2) focus group with CCG stakeholder board; (3) individual interviews with healthcare professionals (HCPs), patients, current and bereaved carers; (4) retrospective cohort study of routinely collected data on EPaCCS usage and (5) data analysis and synthesis of study findings.

Phase one: development of the initial programme theory (June-October 2018)

Phase one is complete and included identification of relevant literature from electronic searches of databases, such as Medline and Google Scholar. The search strategy involved searching for papers which discussed or evaluated shared digital records, for the coordination of palliative care, end of life care, or advance care plans. Reference lists of relevant papers were scanned, and citation searches conducted. Grey literature relating to policy and organisational-based material were sought by searching government and other specialist websites. The lead researcher's own experiences as a General Practitioner (GP) were used as "informed guesswork" (27) and initial meetings were held with key stakeholders, including patients at the local hospice and commissioners. These initial engagements were informal and

For peer review only

were patient and public involvement activities (detailed further below). They did not constitute formal interviews requiring ethics clearance.

The proposed implementation of EPaCCs was broken down and analysed, to understand different elements of this process (Table 1, Column 1). These elements, highlighted as important through literature searches and initial stakeholder engagement, were analysed and detailed into initial CMO statements (Table 1, Column 2). An overview of these CMO statements were then illustrated through a process diagram as illustrated in Figure 2.

At an early stage of the programme theory development, the CMO conjectures were reviewed by, and discussed with, Dr Justin Jagosh, Director of the Centre for Advancement in Realist Evaluation and Synthesis.

The initial programme theory forms a set of hypotheses on what the mechanisms may be, what groups may benefit most or least and the contextual factors that might be important to its success or failure. These hypotheses will be interrogated and tested in phases 2-5 of the study.

Figure 2 - Visual representation of the initial programme theory at a macro level.

Phase two: focus group with CCG stakeholder board (October 2018)

The CCG EOL care board is a multi-disciplinary, multi-organisational system board, whose members are high-level stakeholder representatives from across the CCG, including representatives from community nursing teams, primary care, the ambulance service (which serves a wider geographical area than the CCG area), local hospices, care homes and secondary care. The purpose of the board is to review the current commissioning across all geographical provision, ensuring unified pathways for community, primary and secondary providers to provide consistency for all patients, carers and staff across the system.

For peer review only

Board members will be invited to take part in a focus group following their attendance at the monthly board meeting. Focus groups allow for social interaction, which can help to reveal issues and subsequent points of view that may not be prompted or discovered through individual interviews. This approach will help the study team to gain as wide an understanding as possible of the process of commissioning the EPaCCS and help to refine the initial programme theory.

Participants will be consented to take part in the study prior to the focus group by either LF or LP who will be facilitating. A topic guide will be used to steer the focus group and will enable the research team to test and refine the initial programme theory prior to commencing the in-depth interviews (see table 1).

It is anticipated that the focus group will last approximately 45 minutes and that approximately 5-10 participants will take part in it. The focus group will be audio taped and transcribed verbatim.

Table 1 - Context, mechanism and outcome (CMO) statements that comprise the programme theory, and the questions that will be used, in the focus group with the end-of-life Board, to investigate each CMO statement.

EPaCCs Process	CMOs	Focus group questions
**Commissioning**	1. If the strategy behind the EPaCCs is definable, deliverable and measureable, the aim, purpose and outcomes of EPaCCS will be clear. ***(strategy)***	• How will you evaluate EPaCCS success?<br>• What are the markers of success for you?<br>• What is the CCGs long term vision for the EPaCCS?
	2. If HCPs engage with the EPaCCS positively on early usage and see it as an improvement on any previous EOL register, HCPs will engage positively with EPaCCS. ***(engagement)***	• Given that the previous EOL register was generally not well thought of, or used, how did the CCG plan to get HCPS on board?<br>• How do you think the EPaCCS has been received?
**Commissioning/Uptake/ Adoption**	3. If the EPaCCS is well-publicised and marketed to all stakeholders HCPs will be aware of EPaCCS, understand the aim and purpose of the EPaCCS, and will initiate an EPaCCs template and/or access an EPaCCS record. ***(publicity)***	• How was the EPaCCS publicised and marketed to different groups of healthcare professionals (HCPS)? What are your views on how effective this has been?<br>• How aware do you think HCPs are of EPaCCS and do you think they understand its purpose and importance?
	4. If HCPs receive sufficient support and training, so that they know how to use it, they and will initiate an EPaCCs template and/or access an EPaCCS record. ***(training)***	• Can you tell us about the CCG strategy for providing training and support to different groups of HCPs in the EPaCCS rollout?

		• What do you think about this, and how effective it has been?
**Uptake/Adoption**	5. If HCPs have the time and/or resources to learn a new system, an EPaCCS template will be initiated. ***(time & resources)***	• There are a significant number of GP practices that have not initiated an EPaCCS – do you have any thoughts about why this might be?<br>• Do you think all HCPs will have the time and resources (i.e. they are connected to a computer, have internet and NHS network access) to learn and new system and access EPaCCS?
	6. If HCPs are incentivised to use EPaCCS, an EPaCCS template will be initiated. ***(incentives)***	• Do HCPs have other ways of obtaining the information contained on EPaCCs?<br>• What might these be, and are these ways better or worse, more reliable or less reliable?
**Uptake**	7. If the patient consents to information-sharing and storage of information about their care preferences, an EPaCCS template will be initiated. ***(information sharing)***	• For the EPaCCS to be effective, patients must consent to information-sharing, and the storage of information.<br>• Did you anticipate that this would raise any issues?
	8. If HCPs are near to a computer, are connected to the internet and have access to the GP EMIS record, an EPaCCS template will be initiated. ***(access to system)***	• There is a theory that because EPaCCS is an electronic record, presently only updateable by the GP on EMIS, that this will have an impact on the ability of others to access it and update it and own it. Do you see this as an issue?<br>• What impact do you think this might present?
	9. If HCPs feel able/comfortable having advanced care planning (ACP) conversations with patients, an EPaCCS template will be initiated. ***(ACP conversations)***	• How do you think HCPs feel about having ACP conversations with patients?<br>• Research suggests that patients with non-malignant diagnoses are less likely to be added to EPaCCS.<br>• Do you think this is the case and if so why? Are there other patient groups who might be underrepresented on the EPaCCS?
	10. If HCPs feel that the EPaCCS facilitates, potentially difficult, ACP conversations an EPaCCS template will be initiated. ***(ACP conversations)***	• Some would argue that the EPaCCS template might facilitate ACP conversations with patients – what are your thoughts on this?
	11. If the patient is willing, and has capacity to have ACP conversations, an EPaCCS template will be initiated. ***(ACP conversations)***	• Patients can only record their wishes if they are able to have a conversation with an HCP – what issues do you think this might present?
**Adoption**	12. If EOLC information about a patient can be accessed more efficiently in other ways (i.e. speaking with carer or reading other sources of information) the information on the EPaCCS template may not be accessed. ***(single point of access)***	• Are there any other sources of information that HCPs might access to establish the EOL wishes and needs of a patient and do you think they present an issue of the uptake of EPaCCS?
	13. If HCPs are near to a computer, are connected to the internet and have access to the NHS Network an EPaCCs template will be accessed. ***(access to system)***	• There is a theory that because EPaCCS is an electronic record, presently only updateable by the GP on EMIS, that this will have an impact on the ability of others to access it and update it and own it.<br>• Do you see this as an issue? What impact do you think this might present?
**Adoption/ Care Coordination**	14. If the information does not reflect the current wishes of the patient, care may not be aligned with the patients' preferences. ***(patient preferences)***	• Do you feel that the EPaCCs adequately reflects patient's wishes and preferences for care?
	15. If the patient does not have clear or clinically meetable preferences, or their wishes are subject to frequent change, care may not be aligned with the patient's wishes. ***(patient preferences)***	• Do you feel the EPaCCS adequately reflects the patient's/carer's wishes and preferences regarding end of life care and do you feel these wishes are deliverable? If not, why might this be and what needs to be improved?
**Care Coordination**	16. If HCPs access EPaCCS and consider the information contained within it to be trustworthy (current, relevant, detailed and useful) care will be coordinated by EPaCCs and this care will align with the patient's wishes. ***(trustworthiness of EPaCCS)***	• Do you think the EPaCCS contains all the information HCPs need to enact their patient's wishes and coordinate their patient's care?<br>• Do you consider it to be current, relevant, detailed and useful? If not, why might this be and what needs to be improved?

	17. If EPaCCS does not enhance or improve the care that is already being delivered care may not be coordinated by EPaCCS, consistent or reflect the patients' preferences. ***(patient outcomes)***	• What are your thoughts on the notion that: 'The EPaCCS is not coordinating care, it is simply recording what is already being done'

Phase three: individual interviews with healthcare professionals (HCPs), patients, current and bereaved carers (November 2018 – July 2019)

Healthcare professionals (HCPs)

HCPs from community nursing teams, primary care, the ambulance service, the local hospices, care homes and secondary care will be invited to participate.

GPs working in practices within the CCG area will be invited to support the study and the study team will purposively sample from a list of practices, to include practices that are high-users of EPaCCS and low users of EPaCCS based on data compiled by the CCG.

The research team will purposively sample HCPs who express an interest in participating according to gender, age, and profession to ensure maximum variation in the sample. All interviews with HCPs will take place over the telephone for both pragmatic and methodological reasons. Conducting interviews over the telephone will reduce the time and cost to the study that may be involved in travelling to interviews and well-planned telephone interviews can gather the same material as those held face to face (28).

A topic guide, informed by the evolving programme theory, will be used to ensure consistency across the interviews. This will enable the research team to compare the views of each group at the stage of data analysis. Interviews will last approximately 30 minutes and it is anticipated that approximately 3-5 HCPs will be interviewed from each group (18-30 in total).

Patients, current and bereaved carers

For peer review only

Patients will be approached to take part in interviews through their GP surgeries or the local hospice. The research team will purposively sample from a list of practices, to include practices that are high-users of EPaCCS (20 or more records initiated) and low users of EPaCCS (fewer than 20 records initiated) based on data compiled by the CCG which makes clear the number of EPaCCS templates initiated according to practice. Selected practices will be asked to identify patients, aged 18 and over, receiving EOL care, who they consider might be eligible to take part in the study.

Inclusion criteria:

1. Capacity to give informed consent
2. Aged 18 and over
3. Prognosis 12 months or less as identified by their GP (patient aware of this prognosis)

Potential participants will be sampled purposively to include patients across the age range, with and without an EPaCCS record, with both malignant and non-malignant health conditions.

To recruit patients to the study from the local hospice we will liaise with key clinical staff, who will be responsible for identifying appropriate patients. Once again, potential participants will be sampled purposively to include patients across the age range, with and without an EPaCCS record, with both malignant and non-malignant health conditions.

Alongside their own study information pack, all patients will be given a carer information pack which they can choose to give to their carer if they are happy for their carer to participate in the study.

GPs will also be asked to identify recently bereaved carers (between 8 weeks and 6 months of the death of their relative), who they consider might be eligible to take part in the study. GPs

For peer review only

will be sent details of how to perform an appropriate search within EMIS to identify potential participants.

Interviews with carers will be conducted one-to-one with the interviewer. Interviews with patients will be conducted either one-to-one with the interviewer or in the presence of their carer, according to the wishes of the patient.

Interviews will last approximately 45 minutes and it is anticipated that approximately 15 patients will be interviewed (10 EPaCCS patients and 5 non-EPaCCS patients) and 10 carers (to include both current and bereaved). All interviews will be audio taped and transcribed verbatim.

Phase four: retrospective cohort study of routinely collected data on EPaCCS usage (March 2019 – July 2019)

EMIS data will be accessed to identify all patients, aged 18 and over, who die in the CCG area between 22nd February 2018 and 21st February 2019. Agreements are in place with the CCG to obtain this data. Patients will be identified as either having an EPaCCS record (EPaCCS patient) or not (non-EPaCCS patient), using EMIS coding.

EMIS will be used to characterise both EPaCCS and non-EPaCCS patients in terms of their gender, ethnicity and postcode (as a proxy for socio-economic status according to their Index of Multiple Deprivation Score). Data will be extracted to describe:

1. The proportion of patients that die with an EPaCCS record.
2. When the EPaCCS record is initiated (i.e. how many months/days prior to the patient's death), and by whom.

For peer review only

3. How frequently the EPaCCS record is updated once opened, and who makes any changes.
4. The underlying cause and place of death for EPaCCS and non-EPaCCS patients.
5. The number of hospital admissions and Emergency Department attendances for EPaCCS and non-EPaCCS patients in the last 12 months of life.

Descriptive data will be collected, by the CCG, on EPaCCS usage, including the number of records created by each GP surgery in the CCG area. Data from the integrated digital care record will be accessed to describe which HCPs (GPs, community nurses, hospice HCPs, ambulance HCPs and secondary care clinicians) are accessing these shared end-of-life care records.

Phase five: data analysis and synthesis of study findings (October 2018 – October 2019)

Quantitative methods

Quantitative data will be analysed using Stata v15 and reported using descriptive statistics. Within the context of this realist evaluation, we were keen to use the quantitative data to address a single hypothesis, namely whether nominal possession of an EPaCCS record was associated with increased chance of dying at home. However, logistic regression will be used to determine the adjusted odds ratio and 95% confidence intervals for the associations between having an EPaCCS record and dying at home, considering other factors of interest, including, but not limited to: age, sex, deprivation and underlying cause of death.

Of approximately 8,000 deaths occurring in the CCG area over the year of study, we expect around 10% (800 deaths) of patients to have an EPaCCS (10). If the proportion of deaths occurring at home is expected to be 25% among those without an EPaCCS, we would have

For peer review only

over 99% power to detect an absolute increase of 10% to 35% among those with an EPaCCS. The power would be about 84% if the proportion were increased by 5% to 30%.

Descriptive statistics will be employed to report EPaCCS usage.

Qualitative methods

Data analysis will be conducted using a realist approach informed by Jackson and Kolla's realistic evaluation analysis method (29). This analytic process will involve the following steps:

1. Coding individual units (a discrete C, M or O) within the narratives of the interviews.
2. Identifying the complex connections that link these codes together into dyads or triads.
3. Subsuming the linked codes into themes using thematic analysis (29).

Analysis will begin shortly after data collection starts and be ongoing and iterative. Analysis will inform further data collection: for instance, analytic insights from data gathered in earlier interviews will be used to develop and adapt the programme theory and in turn, identify any changes that need to be made to the topic guide for use during later interviews. The study will generate new programme theories to explain how the EPaCCS works, for whom, and any contextual influences and constraining factors that affect their initiation and usage. Emerging analysis and findings will be discussed with PPI representatives, to explore and clarify findings.

Qualitative and quantitative data will be collected concurrently, giving equal weight to each (30). Data will be triangulated in order to test and refine the programme theories, accepting that any findings are fallible and with time and further study new data are bound to emerge (31). The synthesised study findings will establish the potential outcomes of EPaCCS, identify the underlying mechanisms which explain how they produce these effects and

For peer review only

highlight the key contextual factors that affect their success or failure. Recommendations can then be made for the development and implementation of EPaCCS.

Patient and Public Involvement

To support the development of this study protocol, members of the study team (LF and LP) presented and discussed an outline proposal of this study to patients, staff and carers at the local hospice on two separate occasions in April 2018. Approximately 10 participants voluntarily took part in these, semi-structured, discussions, in which we asked specific questions concerning ethical and methodological issues. Participants were also encouraged to ask any questions. These meetings raised several important issues which have been incorporated into the design of this study. Such issues included allowing patients the choice of whether to have a carer sit alongside them during their interview and which HCPs they felt it was important that the study team spoke to, due to the involvement they had in providing care for patients. The meetings also discussed what terms, wording and questions would be acceptable to patients and carers to read and hear in the study information documents and interviews.

At the end of both meetings, patients and carers were invited to continue to support the design of the study should they wished. Two members came forward expressing a wish to be more actively involved in the study. They have kindly been involved in reviewing all the study literature, including the topic guides, study information sheets and the lay summary for this protocol. It is hoped that they will wish to continue their involvement with the study. This will include informing the content of materials for lay audiences, drawing links to groups and forums that the research team may be unaware of, and supporting the study team with the interpretation and dissemination of study findings. To ensure ongoing PPI, all patients and carers taking part in the study will be invited to support the ongoing development of the study.

For peer review only

Ethics and dissemination

The study has been approved by NHS South West – Frenchay Research Ethics Committee (REC reference number: 18/SW/0198).

The research team will disseminate the findings to a range of stakeholders. We will draw on the networks and expertise of the local CCG end-of-life care board to disseminate the research outputs widely and appropriately. Key audiences include patient and carer organisations, GPs and community nursing teams in primary care, ambulatory services and care home staff, HCPs working in secondary palliative care services and hospices, managers and directors within healthcare organisations with responsibility to provide high-quality services within budget and healthcare policymakers, nationally and internationally.

Acknowledgements

We would like to thank all the patients, carers and HCPs who helped to support the PPI in development of this study.

We would also like to thank Dr Justin Jagosh, Director of the Centre for Advancement in Realist Evaluation and Synthesis (https://realistmethodology-cares.org/), for his support and guidance in developing the initial programme theory.

Contributors

LP was the principal investigator for this qualitative study. LF, MF, RM and SP all contributed to the development of the study protocol. LP and LF drafted this manuscript. All authors contributed to the editing of the final manuscript and the refining of its intellectual content.

For peer review only

Funding

LP's time is paid for by a NIHR School for Primary Care Research GP Career Progression Fellowship. This fellowship funding will also meet the research costs. LF's time is paid for by NIHR Research Capability Funding. MF's time is supported by the NIHR Collaboration for Leadership in Applied Health Research and Care West (NIHR CLAHRC West). The study is supported by the NIHR West of England Clinical Research Network. The views expressed in this publication are those of the authors and not necessarily those of the NHS, the National Institute for Health Research or the Department of Health and Social Care.

Competing interests

We have read and understood BMJ policy on declaration of interests and declare that we have no competing interests.

References

1. National Institute for Health and Care Excellence (2004). Improving supportive and palliative care for adults with cancer. Cancer service guideline [CSG4].
2. Palliative and end of life care Priority Setting Partnership: Putting patients, carers and clinicians at the heart of palliative and end of life care research. January 2015. Available at: https://www.mariecurie.org.uk/globalassets/media/documents/research/PeolcPSP_Final_Report.pdf (accessed 30/05/18)
3. Barker I, Steventon A, Deeny SR. Association between continuity of care in general practice and hospital admissions for ambulatory care sensitive conditions: cross sectional study of routinely collected, person level data. *BMJ* 2017;356:j84

For peer review only

4. Schweitzer B, Blankenstein N, Deliens L, Van Der Horst H. Out-of-hours palliative care provided by GP co-operatives in the Netherlands: A focus group study. *European Journal of General Practice*, 2011; 17: 160–166.
5. Hall S, Murchie P, Campbell C and Murray SA. Introducing an electronic Palliative Care Summary (ePCS) in Scotland: patient, carer and professional perspectives. *Family Practice* 2012; 29:576–585.
6. Richards SH, Winder R, Seamark C, Seamark D, Avery S. Gilbert J, Barwick A and Campbell JL. 2011: The experiences and needs of people seeking palliative health care out-of-hours: a qualitative study. *Primary Health Care Research & Development* 12(2), 165–78; available from: PM:21457601.
7. Leydon GM, Shergill NK, Campion-Smith C, et al. Discontinuity of care at end of life: a qualitative exploration of OOH end of life care. *BMJ Supportive & Palliative Care* 2013;3:412-421.
8. Purdy S, Lasseter G, Griffin T, Wye L. Impact of the Marie Curie Cancer Care Delivering Choice Programme in Somerset and North Somerset on place of death and hospital usage: a retrospective cohort study. *BMJ Supportive & Palliative Care* 2014;0:1–6.
9. Asprey A, Richards SH, Wright C, Seamark C, Seamark D and Moxon J. Transferring information to an out-of-hours primary care service for patients with palliative care needs: an action research study to improve the use of handover forms. *Primary Health Care Research & Development* 2013; 14: 7–20.
10. Wye L, Lasseter G, Simmonds B, Duncan L, Percival J and Purdy S. Electronic palliative care coordinating systems (EPaCCS) may not facilitate home deaths: A mixed methods evaluation of end of life care in two English counties. *Journal of Research in Nursing*, 2016, Vol. 21(2) 96–107

For peer review only

11. National Institute for Health and Clinical Excellence (2011) End of Life Care for Adults. NICE Quality Standard [QS13]

12. Petrova M, Riley J, Abel J and Barclay S. Crash course in EPaCCS (Electronic Palliative Care Coordination Systems): 8 years of successes and failures in patient data sharing to learn from. *BMJ Supportive & Palliative Care* 2016;0:1–9.

13. Ali AA, Adam R, Taylor D, et al. Use of a structured palliative care summary in patients with established cancer is associated with reduced hospital admissions by out-of-hours general practitioners in Grampian. BMJ Support Palliat Care2013;3:452–5.doi:10.1136/bmjspcare-2012-000371

14. Turner V, Flemming K. Socioeconomic factors affecting access to preferred place of death: A qualitative evidence synthesis. *Palliative medicine* 2019 Mar 8:0269216319835146.

15. Department of Health (2016) Our Commitment to you for end of life care: the Government Response to the Review of Choice in End of Life Care. Available at: https://assets.publishing.service.gov.uk/government/uploads/system/uploads/attachment_data/file/536326/choice-response.pdf (accessed 21/03/19)

16. Office for National Statistics. National Survey of Bereaved People (VOICES): England, 2015. Available at: https://www.ons.gov.uk/peoplepopulationandcommunity/healthandsocialcare/healthcaresystem/bulletins/nationalsurveyofbereavedpeoplevoices/england2015#preferences-and-choice-at-the-end-of-life (accessed 30/05/18)

17. Public Health England, End of Life Care Profiles. Available at: https://fingertips.phe.org.uk/profile/end-of-life (accessed 30/05/18)

For peer review only - http://bmjopen.bmj.com/site/about/guidelines.xhtml

18. Allsop MJ, Kite S, McDermott S, Penn N, Millares-Martin P and Bennett MI. Electronic palliative care coordination systems: Devising and testing a methodology for evaluating documentation, *Palliative Medicine* 2017 May; 31(5): 475–482.

19. Fallowfield L and Jenkins V. Communicating sad, bad, and difficult news in medicine. *The Lancet*, 2004;*363*(9405), 312-319.

20. Gibbins J, McCoubrie R, and Forbes K. Why are newly qualified doctors unprepared to care for patients at the end of life? *Medical education*;2011, *45*(4), 389-399.

21. Whole Systems Partnership. (2016) *Independent evaluation of Electronic Palliative Care Co-ordination Systems (EPaCCS) in England: Final Report*, March 2016

22. Wye L, Lasseter GM, Percival JF, Simmonds BAJ, Duncan LJ, Purdy S. Independent Evaluation of the Marie Curie Cancer Care Delivering Choice Programme in Somerset and North Somerset. Centre for Primary Health Care, Department of Social and Community Medicine, The University of Bristol, 2012.

23. NHS Digital (2015). SCCI1580: Palliative Care Co-ordination: Core Content. Available at: https://digital.nhs.uk/data-and-information/information-standards/information-standards-and-data-collections-including-extractions/publications-and-notifications/standards-and-collections/scci1580-palliative-care-co-ordination-core-content (accessed 23/01/19)

24. Pawson R and Tilley N. Realistic Evaluation. London: Sage, 2005.

25. National End of Life Care Intelligence Network. (2014). *Electronic Palliative Care Co-ordination Systems (EPaCCS) in England: Survey of clinical commissioning groups (2013).*

26. Pawson R. Evidence based policy: A realist perspective. London: Sage, 2006.

For peer review only

27. Greenhalgh T, Wong G, Jagosh J, et al Protocol—the RAMESES II study: developing guidance and reporting standards for realist evaluation BMJ Open 2015;5:e008567. doi: 10.1136/bmjopen-2015-008567

28. Sturges JE, Hanrahan KJ. Comparing telephone and face-to-face qualitative interviewing: a research note. Qual Res. 2004 Apr;4(1):107-18.

29. Jackson SF, Kolla G. A new realistic evaluation analysis method: linked coding of context, mechanism, and outcome relationships. American Journal of Evaluation. 2012 Sep;33(3):339-49.

30. Hanson WE, Creswell JW, Clark VL, Petska KS, Creswell JD. Mixed methods research designs in counseling psychology. Journal of counseling psychology. 2005 Apr;52(2):224.

31. Emmel N, Greenhalgh J, Manzano A, Monaghan M, Dalkin S (Eds). Doing realist research. London: Sage, 2018.

For peer review only

Figure 1 – Definition of Context, Mechanism and Outcome

Context

Something that existed prior to the introduction of the EPaCCS, for example cultural views and beliefs around talking about death and dying and Advanced Care Planning (ACP) conversations. Contexts might also refer to the setting, (e.g. primary or secondary care) or patient characteristics (e.g. underlying diagnosis, socio-demographics, mental capacity).

Mechanism

The intended or unintended resources created by an intervention and the response to those resources (cognitive, emotional, motivational etc) by Healthcare Professionals (HCPs), patients and carers. Mechanisms can pertain to why HCPs and patients choose (or choose not) to utilise the EPaCCS.

Outcome

An outcome will define the result of the EPaCCS whether intended (did the project succeed against the criteria it set itself at the outset), and also the unplanned and/or unexpected impacts.

Informed by (24) and (26).

For peer review only

Figure 2 – Visual representation of the initial programme theory at a macro level. See table 1 for associated CMO statements.

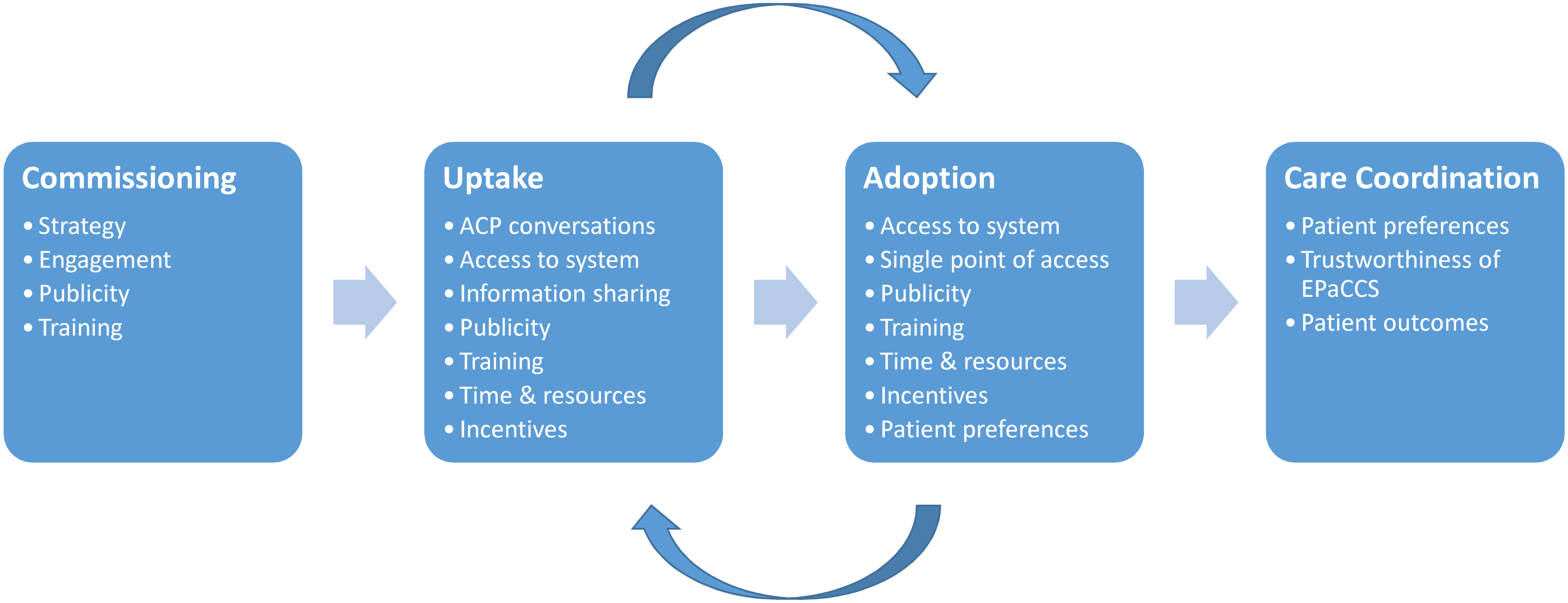

BMJ Open

THE IMPACT OF ELECTRONIC PALLIATIVE CARE COORDINATION SYSTEMS (EPaCCS) ON CARE AT THE END OF LIFE ACROSS MULTIPLE CARE SECTORS, IN ONE CLINICAL COMMISSIONING GROUP AREA, IN ENGLAND: A REALIST EVALUATION PROTOCOL

Journal:	*BMJ Open*
Manuscript ID	bmjopen-2019-031153.R3
Article Type:	Protocol
Date Submitted by the Author:	23-Jan-2020
Complete List of Authors:	Pocock, Lucy; University of Bristol, Centre for Academic Primary Care<br>French, Lydia; University of Bristol, Centre for Academic Primary Care<br>Farr, Michelle; University of Bristol, NIHR CLAHRC West, Bristol Medical School<br>Morris, Richard; University of Bristol, School of Social & Community Medicine<br>Purdy, Sarah; University of Bristol, Centre for Academic Primary Care
**Primary Subject Heading**:	Palliative care
Secondary Subject Heading:	Health services research
Keywords:	PALLIATIVE CARE, Quality in health care < HEALTH SERVICES ADMINISTRATION & MANAGEMENT, PRIMARY CARE, QUALITATIVE RESEARCH

SCHOLARONE™
Manuscripts

I, the Submitting Author has the right to grant and does grant on behalf of all authors of the Work (as defined in the below author licence), an exclusive licence and/or a non-exclusive licence for contributions from authors who are: i) UK Crown employees; ii) where BMJ has agreed a CC-BY licence shall apply, and/or iii) in accordance with the terms applicable for US Federal Government officers or employees acting as part of their official duties; on a worldwide, perpetual, irrevocable, royalty-free basis to BMJ Publishing Group Ltd ("BMJ") its licensees and where the relevant Journal is co-owned by BMJ to the co-owners of the Journal, to publish the Work in this journal and any other BMJ products and to exploit all rights, as set out in our licence.

The Submitting Author accepts and understands that any supply made under these terms is made by BMJ to the Submitting Author unless you are acting as an employee on behalf of your employer or a postgraduate student of an affiliated institution which is paying any applicable article publishing charge ("APC") for Open Access articles. Where the Submitting Author wishes to make the Work available on an Open Access basis (and intends to pay the relevant APC), the terms of reuse of such Open Access shall be governed by a Creative Commons licence – details of these licences and which Creative Commons licence will apply to this Work are set out in our licence referred to above.

Other than as permitted in any relevant BMJ Author's Self Archiving Policies, I confirm this Work has not been accepted for publication elsewhere, is not being considered for publication elsewhere and does not duplicate material already published. I confirm all authors consent to publication of this Work and authorise the granting of this licence.

For peer review only

THE IMPACT OF ELECTRONIC PALLIATIVE CARE COORDINATION SYSTEMS (EPaCCS) ON CARE AT THE END OF LIFE ACROSS MULTIPLE CARE SECTORS, IN ONE CLINICAL COMMISSIONING GROUP AREA, IN ENGLAND: A REALIST EVALUATION PROTOCOL

Corresponding author

Lucy V Pocock, Population Health Sciences, Bristol Medical School, University of Bristol, Canynge Hall, 39 Whatley Road, Bristol, BS8 2PS

Lucy.pocock@bristol.ac.uk, Tel: 07810 002320

Co-authors

Lydia R M French, Population Health Sciences, Bristol Medical School, University of Bristol, Bristol, UK

Michelle Farr, The National Institute for Health Research Collaboration for Leadership in Applied Health Research and Care West (NIHR CLAHRC West) at University Hospitals Bristol NHS Foundation Trust, UK

Richard Morris, Population Health Sciences, Bristol Medical School, University of Bristol, Bristol, UK

Sarah Purdy, Population Health Sciences, Bristol Medical School, University of Bristol, Bristol, UK,

Word Count: 3862

For peer review only

Abstract

Introduction: Electronic palliative care coordination systems (EPaCCS) aim to support people approaching the end-of life to receive consistent care, according to their wishes, that is coordinated effectively across multiple care sectors. They are in use across the UK although empirical evidence into their effectiveness is poor. This paper presents a protocol of a mixed-methods study, to understand how, and by whom, EPaCCS are being used and whether EPaCCS are enabling Healthcare Professionals (HCPs) to coordinate patients' end-of-life (EOL) care.

Methods and Analysis: This is a mixed-methods study, carried out within a realist paradigm, to evaluate the impact of an EPaCCS on EOL care as provided by a Clinical Commissioning Group (CCG) in England. This study has two aims: 1. Describe the socio-demographic characteristics of patients who die with an EPaCCS record, their underlying cause of death and place of death and compare these with patients who die without an EPaCCS record. 2. Explore the impact of an EPaCCS on the experience of receiving EOL care for patients and their carers, and understand HCPs' views and experiences of utilising an EPaCCS to coordinate care for their patients. The study will be conducted in five phases: (1) development of the initial programme theory; (2) focus group with CCG stakeholder board; (3) individual interviews with HCPs, patients, current and bereaved carers; (4) retrospective cohort study of routinely collected data on EPaCCS usage and (5) data analysis and synthesis of study findings.

Ethics and dissemination: The study has been approved by NHS South West – Frenchay Research Ethics Committee (REC reference number: 18/SW/0198). Findings will be published in a wide range of outputs targeted at key audiences.

Strengths and limitations of this study

For peer review only

- Using a theory-driven realist evaluation approach, findings from this study are expected to generate contextually relevant evidence for other care coordination systems.
- The study will test and refine these theories using a mixed methods approach, enhancing the credibility of the evaluation findings.
- This study addresses the need for qualitative research into the use of EPaCCS, offering much needed insight into patient and carers' experiences of EPaCCS.
- The study will investigate the impact of only one of the many EPaCCS developed and implemented in the UK.
- The qualitative component has a potentially small sample size, however, the aim of the study is not to find a robust causal mechanism for EPaCCS, but to unpack the contexts and mechanisms that work in certain circumstances.

Key words

Palliative Care, Quality in Health Care, Primary Care, Qualitative Research

For peer review only

Background

People at the end-of-life (taken here to mean in the last 12 months of life) frequently receive care from a wide variety of teams and organisations. Much of this care is accessed in the out-of-hours period (overnight and at the weekend), when they are unlikely to see a Healthcare Professional (HCP) who knows them well. Out-of-hours provision of palliative care (defined by NICE as “the active holistic care of patients with advanced, progressive illness” (1)) has been identified as a key priority for future research by the Palliative and End of Life Care Priority Setting Partnership, initiated in 2013 by Marie Curie (2). This process identified the top 10 unanswered research questions and the question with the highest priority was, “What are the best ways of providing palliative care outside of working hours to avoid crises and help patients to stay in their place of choice?”

Continuity of care is important for anyone with complex health and social care needs, but particularly for those at the end-of-life (EOL) (3). Until recently HCPs have communicated patients’ end of life care plans, to other HCPs, by means of a variety of methods, including shared end of life care registers, letters, faxes and telephone and/or face to face conversations. Despite this, a lack of information sharing has been repeatedly cited as a barrier to the provision of good quality EOL care outside of normal working hours (4,5,6). Recent studies looking at the experiences and needs of people seeking palliative care out-of-hours found that most patients expect the HCP to be able to access a summary of their complex medical history and many voiced concerns that their full record could not be accessed by out-of-hours clinicians (5,7). For some patients, lack of access to their notes is a deterrent to accessing care out-of-hours (6).

Nationally, the policy drive to address the issue of information continuity has resulted in the development of Electronic Palliative Care Coordination Systems, or EPaCCS (5,8,9,10). These records are usually completed by the patients’ GP or community nurse, including

For peer review only

patients' advance care wishes, and are accessible across multiple care sectors (10). The purpose of EPaCCS is to provide a shared local record for health and social care professionals, with key information about an individual approaching the EOL, including their expressed preferences for care. In accordance with the Quality Statements in the NICE End of Life Care Standard for Adults (11) the intent is for EPaCCS to support people approaching the EOL to receive consistent care that is coordinated effectively across all relevant settings.

An EPaCCS record can take various forms, including web-based electronic registers, systems based on sharing care summaries or care plans, alongside patients' electronic records. They store a dynamic record of a patient's medical condition, treatment, wishes and preferences, and provide information about the medication a patient is receiving, contact details of any carers, and services involved in providing care and support to the patient.

Sharing information about patients' EOL care has the potential to improve coordination and communication across care settings (12). It may reduce the chance of emergency department attendance, hospital admission and dying in hospital (8, 13).

It is now recognised that place of death is unlikely to be the most important factor in achieving a good death and a recent UK study has proposed that it is the presence of loved ones that is more important than the physical location (14). However, death in preferred place remains a significant measure of quality of death (15) and, according to the Voices survey of bereaved people, despite 81% of respondents believing that the deceased had wanted to die at home less than a quarter of people actually achieve this (16, 17).

Quantitative studies have shown striking differences in place of death with EPaCCS but are potentially biased and confounded (12). A more recent study challenges these assumptions, suggesting that the increase in home deaths could in fact be due to selection bias (few secondary care colleagues used the systems and therefore hospital deaths are not captured)

For peer review only

(10). The findings of this study also underscore the importance of qualitative approaches, which can offer crucial insights into what is happening on the ground, away from broad claims of EPaCCS benefits arrived at solely through quantitative methods. Without understanding the experiences of patients and carers, together with the perspectives of HCPs, it is difficult to evaluate the effectiveness of EPaCCS (18).

Technology in isolation is not guaranteed to bring benefit and the initiation of an EPaCCS relies on healthcare professionals initiating conversations about death and dying. There is evidence that these conversations are difficult for HCPs (19), with many choosing to avoid the conversation altogether (20). What impact EPaCCS has upon these conversations, if any, is not clear.

A recent independent evaluation of EPaCCS found that “it was not possible to demonstrate that EPaCCS was making any difference to the care patients were receiving at the end-of-life because the range of clients for whom EPaCCS was being used remains focused on cancer, and the ability of EPaCCS systems to report on progress and outcomes remains generally poor” (21).

We therefore do not know if EPaCCS acts to improve practice or whether it simply documents and reflects what is already taking place in practice (21). Indeed, the need to gather evidence of effectiveness of EPaCCS is vitally important, as it has already been widely and uncritically adopted by the NHS.

To summarise, very little research has been carried out to understand how, and by whom, EPaCCS are being used and, perhaps more importantly, whether EPaCCS are enabling HCPs to support patients’ EOL wishes. Rigorous evaluation and research are urgently needed to investigate to what extent EPaCCS influence services working together to support ‘a good death’, the outcome that stakeholders think is of most importance (22).

For peer review only

Aims, Objectives and Research Questions

The study has two aims. These are to:

1. Describe the socio-demographic characteristics of patients who die with an EPaCCS record, their underlying cause of death and place of death and compare these with patients who die without an EPaCCS record.

2. Explore the impact of an EPaCCS on the experience of receiving EOL care for patients and their carers, and understand HCPs' views and experiences of utilising an EPaCCS to provide palliative care to their patients.

Project methodology

Study setting

This study will be implemented in England, where one Clinical Commissioning Group (CCG) has recently developed and rolled out an EPaCCS. This involved the dissemination of an EMIS template, which was circulated to General Practices, to help ensure consistent data entry and coding. EMIS is the most widely used primary care clinical system in the UK and allows real-time patient information to be shared securely between different organisations. All practices in the CCG area are EMIS users and it is now also used by the local hospice and many of the community nursing teams.

The EPaCCS template was developed following extensive local clinical consultation and the National Information Standard (23). Although some organisations within the CCG area (ambulance service, secondary and social care) are non-EMIS users, information from the EPaCCS template can be viewed across the local health community, via the integrated digital care record used by health and social care professionals in the CCG area, which went live at the end of February 2018. The integrated digital care record contains some of the information

held at GP practices, hospital departments, community services, mental health trusts, out of hours services and local authorities across the CCG area, combining it into a single, shared digital record.

Conceptual framework

This study will draw on a realist evaluation approach (24). A randomised, experimental study design is not possible as the implementation of EPaCCS has been strongly advocated and promoted by NHS England, with 83% of Clinical Commissioning Groups (CCGs) in England reported to have an operational EPaCCS, or be in the planning stages, by 2013 (25). The CCG had recently operationalised an EPaCCS at the time of study design.

By their nature, EPaCCS involve multiple organisations and a multidisciplinary style of work. It therefore requires a novel methodological approach to evaluation as described in this protocol.

Realist evaluation is a theory-driven approach designed for evaluating complex interventions, such as EPaCCS, where the outcomes are influenced by the way the intervention is delivered and in what context. Due to this complexity, any evaluation of EPaCCS seeking to determine linear causal relationships or simply find out if the intervention "works" is unlikely to be useful. Pawson and Tilley (24), the developers of realistic evaluation, suggest that the results of an intervention (outcomes) are dependent on the introduction of appropriate reasoning and resources (mechanisms) and how these then interact with existing social and cultural condition (contexts). For the purposes of this study, we have defined context (C), mechanism (M) and outcome (O) in Figure 1.

Figure 1 – Definition of Context, Mechanism and Outcome

A realist approach acknowledges that complex interventions only ever work for certain people, in particular circumstances. The key task of a realist evaluation is to understand and

For peer review only

explain these patterns by asking the exploratory question: *what works, for whom and in what circumstances?*

According to Pawson, interventions or "programmes", such as the EPaCCS, are 'theories incarnate' and every programme has a theoretical underpinning (26). Before conducting a realist evaluation, the researchers must develop a theory, or theories, that explain what works, for whom, under what circumstances and how. This is sometimes known as the "initial programme theory" and is described through CMO conjectures. This theory, or theories, are then tested through the process of the evaluation.

The study will be conducted in five phases: (1) development of the initial programme theory; (2) focus group with CCG stakeholder board; (3) individual interviews with healthcare professionals (HCPs), patients, current and bereaved carers; (4) retrospective cohort study of routinely collected data on EPaCCS usage and (5) data analysis and synthesis of study findings.

Phase one: development of the initial programme theory (June-October 2018)

Phase one is complete and included identification of relevant literature from electronic searches of databases, such as Medline and Google Scholar. The search strategy involved searching for papers which discussed or evaluated shared digital records, for the coordination of palliative care, end of life care, or advance care plans. Reference lists of relevant papers were scanned, and citation searches conducted. Grey literature relating to policy and organisational-based material were sought by searching government and other specialist websites. The lead researcher's own experiences as a General Practitioner (GP) were used as "informed guesswork" (27) and initial meetings were held with key stakeholders, including patients at the local hospice and commissioners. These initial engagements were informal and

For peer review only

were patient and public involvement activities (detailed further below). They did not constitute formal interviews requiring ethics clearance.

The proposed implementation of EPaCCs was broken down and analysed, to understand different elements of this process (Table 1, Column 1). These elements, highlighted as important through literature searches and initial stakeholder engagement, were analysed and detailed into initial CMO statements (Table 1, Column 2). An overview of these CMO statements were then illustrated through a process diagram as illustrated in Figure 2.

At an early stage of the programme theory development, the CMO conjectures were reviewed by, and discussed with, Dr Justin Jagosh, Director of the Centre for Advancement in Realist Evaluation and Synthesis.

The initial programme theory forms a set of hypotheses on what the mechanisms may be, what groups may benefit most or least and the contextual factors that might be important to its success or failure. These hypotheses will be interrogated and tested in phases 2-5 of the study.

Figure 2 - Visual representation of the initial programme theory at a macro level.

Phase two: focus group with CCG stakeholder board (October 2018)

The CCG EOL care board is a multi-disciplinary, multi-organisational system board, whose members are high-level stakeholder representatives from across the CCG, including representatives from community nursing teams, primary care, the ambulance service (which serves a wider geographical area than the CCG area), local hospices, care homes and secondary care. The purpose of the board is to review the current commissioning across all geographical provision, ensuring unified pathways for community, primary and secondary providers to provide consistency for all patients, carers and staff across the system.

For peer review only - http://bmjopen.bmj.com/site/about/guidelines.xhtml

Board members will be invited to take part in a focus group following their attendance at the monthly board meeting. Focus groups allow for social interaction, which can help to reveal issues and subsequent points of view that may not be prompted or discovered through individual interviews. This approach will help the study team to gain as wide an understanding as possible of the process of commissioning the EPaCCS and help to refine the initial programme theory.

Participants will be consented to take part in the study prior to the focus group by either LF or LP who will be facilitating. A topic guide will be used to steer the focus group and will enable the research team to test and refine the initial programme theory prior to commencing the in-depth interviews (see table 1).

It is anticipated that the focus group will last approximately 45 minutes and that approximately 5-10 participants will take part in it. The focus group will be audio taped and transcribed verbatim.

Table 1: Programme theory for the EPaCCs study, comprising the 17 context, mechanism and outcome (CMO) statements that inform the programme theory, and the questions that will be used, in the focus group with the end-of-life Board, to investigate each CMO statement.

EPaCCs Process	CMOs	Focus group questions
**Commissioning**	1. If the strategy behind the EPaCCs is definable, deliverable and measureable, the aim, purpose and outcomes of EPaCCS will be clear. ***(strategy)***	• How will you evaluate EPaCCS success?<br>• What are the markers of success for you?<br>• What is the CCGs long term vision for the EPaCCS?
	2. If HCPs engage with the EPaCCS positively on early usage and see it as an improvement on any previous EOL register, HCPs will engage positively with EPaCCS. ***(engagement)***	• Given that the previous EOL register was generally not well thought of, or used, how did the CCG plan to get HCPS on board?<br>• How do you think the EPaCCS has been received?
**Commissioning/Uptake/Adoption**	3. If the EPaCCS is well-publicised and marketed to all stakeholders HCPs will be aware of EPaCCS, understand the aim and purpose of the EPaCCS, and will initiate an EPaCCs template and/or access an EPaCCS record. ***(publicity)***	• How was the EPaCCS publicised and marketed to different groups of healthcare professionals (HCPS)? What are your views on how effective this has been?<br>• How aware do you think HCPs are of EPaCCS and do you think they understand its purpose and importance?
	4. If HCPs receive sufficient support and training, so that they know how to use it, they and will initiate an EPaCCs template	• Can you tell us about the CCG strategy for providing training and support to different groups of HCPs in the EPaCCS rollout?

	and/or access an EPaCCS record. ***(training)***	• What do you think about this, and how effective it has been?
**Uptake/Adoption**	5. If HCPs have the time and/or resources to learn a new system, an EPaCCS template will be initiated. ***(time & resources)***	• There are a significant number of GP practices that have not initiated an EPaCCS – do you have any thoughts about why this might be?<br>• Do you think all HCPs will have the time and resources (i.e. they are connected to a computer, have internet and NHS network access) to learn and new system and access EPaCCS?
	6. If HCPs are incentivised to use EPaCCS, an EPaCCS template will be initiated. ***(incentives)***	• Do HCPs have other ways of obtaining the information contained on EPaCCs?<br>• What might these be, and are these ways better or worse, more reliable or less reliable?
**Uptake**	7. If the patient consents to information-sharing and storage of information about their care preferences, an EPaCCS template will be initiated. ***(information sharing)***	• For the EPaCCS to be effective, patients must consent to information-sharing, and the storage of information.<br>• Did you anticipate that this would raise any issues?
	8. If HCPs are near to a computer, are connected to the internet and have access to the GP EMIS record, an EPaCCS template will be initiated. ***(access to system)***	• There is a theory that because EPaCCS is an electronic record, presently only updateable by the GP on EMIS, that this will have an impact on the ability of others to access it and update it and own it. Do you see this as an issue?<br>• What impact do you think this might present?
	9. If HCPs feel able/comfortable having advanced care planning (ACP) conversations with patients, an EPaCCS template will be initiated. ***(ACP conversations)***	• How do you think HCPs feel about having ACP conversations with patients?<br>• Research suggests that patients with non-malignant diagnoses are less likely to be added to EPaCCS.<br>• Do you think this is the case and if so why? Are there other patient groups who might be underrepresented on the EPaCCS?
	10. If HCPs feel that the EPaCCS facilitates, potentially difficult, ACP conversations an EPaCCS template will be initiated. ***(ACP conversations)***	• Some would argue that the EPaCCS template might facilitate ACP conversations with patients – what are your thoughts on this?
	11. If the patient is willing, and has capacity to have ACP conversations, an EPaCCS template will be initiated. ***(ACP conversations)***	• Patients can only record their wishes if they are able to have a conversation with an HCP – what issues do you think this might present?
**Adoption**	12. If EOLC information about a patient can be accessed more efficiently in other ways (i.e. speaking with carer or reading other sources of information) the information on the EPaCCS template may not be accessed. ***(single point of access)***	• Are there any other sources of information that HCPs might access to establish the EOL wishes and needs of a patient and do you think they present an issue of the uptake of EPaCCS?
	13. If HCPs are near to a computer, are connected to the internet and have access to the NHS Network an EPaCCs template will be accessed. ***(access to system)***	• There is a theory that because EPaCCS is an electronic record, presently only updateable by the GP on EMIS, that this will have an impact on the ability of others to access it and update it and own it.<br>• Do you see this as an issue? What impact do you think this might present?
**Adoption/Care Coordination**	14. If the information does not reflect the current wishes of the patient, care may not be aligned with the patients' preferences. ***(patient preferences)***	• Do you feel that the EPaCCs adequately reflects patient's wishes and preferences for care?
	15. If the patient does not have clear or clinically meetable preferences, or their wishes are subject to frequent change, care may not be aligned with the patient's wishes. ***(patient preferences)***	• Do you feel the EPaCCS adequately reflects the patient's/carer's wishes and preferences regarding end of life care and do you feel these wishes are deliverable? If not, why might this be and what needs to be improved?

**Care Coordination**	16. If HCPs access EPaCCS and consider the information contained within it to be trustworthy (current, relevant, detailed and useful) care will be coordinated by EPaCCs and this care will align with the patient's wishes. ***(trustworthiness of EPaCCS)***	• Do you think the EPaCCS contains all the information HCPs need to enact their patient's wishes and coordinate their patient's care?<br>• Do you consider it to be current, relevant, detailed and useful? If not, why might this be and what needs to be improved?
	17. If EPaCCS does not enhance or improve the care that is already being delivered care may not be coordinated by EPaCCS, consistent or reflect the patients' preferences. ***(patient outcomes)***	• What are your thoughts on the notion that: 'The EPaCCS is not coordinating care, it is simply recording what is already being done'

Phase three: individual interviews with healthcare professionals (HCPs), patients, current and bereaved carers (November 2018 – July 2019)

Healthcare professionals (HCPs)

HCPs from community nursing teams, primary care, the ambulance service, the local hospices, care homes and secondary care will be invited to participate.

GPs working in practices within the CCG area will be invited to support the study and the study team will purposively sample from a list of practices, to include practices that are high-users of EPaCCS and low users of EPaCCS based on data compiled by the CCG.

The research team will purposively sample HCPs who express an interest in participating according to gender, age, and profession to ensure maximum variation in the sample. All interviews with HCPs will take place over the telephone for both pragmatic and methodological reasons. Conducting interviews over the telephone will reduce the time and cost to the study that may be involved in travelling to interviews and well-planned telephone interviews can gather the same material as those held face to face (28).

A topic guide, informed by the evolving programme theory, will be used to ensure consistency across the interviews. This will enable the research team to compare the views of each group at the stage of data analysis. Interviews will last approximately 30 minutes and it

For peer review only

is anticipated that approximately 3-5 HCPs will be interviewed from each group (18-30 in total).

Patients, current and bereaved carers

Patients will be approached to take part in interviews through their GP surgeries or the local hospice. The research team will purposively sample from a list of practices, to include practices that are high-users of EPaCCS and low users of EPaCCS. High EPaCCS use will be defined as practices that have created greater than 20 EPaCCS records (the median number of records across all practices) 4 months post-implementation, based on data extracted by the CCG. Selected practices will be asked to identify patients, aged 18 and over, receiving EOL care, who they consider might be eligible to take part in the study.

Inclusion criteria:

1. Capacity to give informed consent
2. Aged 18 and over
3. Prognosis 12 months or less as identified by their GP (patient aware of this prognosis)

Potential participants will be sampled purposively to include patients across the age range, with and without an EPaCCS record, with both malignant and non-malignant health conditions.

To recruit patients to the study from the local hospice we will liaise with key clinical staff, who will be responsible for identifying appropriate patients. Once again, potential participants will be sampled purposively to include patients across the age range, with and without an EPaCCS record, with both malignant and non-malignant health conditions.

For peer review only

Alongside their own study information pack, all patients will be given a carer information pack which they can choose to give to their carer if they are happy for their carer to participate in the study.

GPs will also be asked to identify recently bereaved carers (between 8 weeks and 6 months of the death of their relative), who they consider might be eligible to take part in the study. GPs will be sent details of how to perform an appropriate search within EMIS to identify potential participants.

Interviews with carers will be conducted one-to-one with the interviewer. Interviews with patients will be conducted either one-to-one with the interviewer or in the presence of their carer, according to the wishes of the patient.

Interviews will last approximately 45 minutes and it is anticipated that approximately 15 patients will be interviewed (10 EPaCCS patients and 5 non-EPaCCS patients) and 10 carers (to include both current and bereaved). All interviews will be audio taped and transcribed verbatim.

Phase four: retrospective cohort study of routinely collected data on EPaCCS usage (March 2019 – July 2019)

EMIS data will be accessed to identify all patients, aged 18 and over, who die in the CCG area between 22nd February 2018 and 21st February 2019. Agreements are in place with the CCG to obtain this data. Patients will be identified as either having an EPaCCS record (EPaCCS patient) or not (non-EPaCCS patient), using EMIS coding.

EMIS will be used to characterise both EPaCCS and non-EPaCCS patients in terms of their gender, ethnicity and postcode (as a proxy for socio-economic status according to their Index of Multiple Deprivation Score). Data will be extracted to describe:

For peer review only

1. The proportion of patients that die with an EPaCCS record.
2. When the EPaCCS record is initiated (i.e. how many months/days prior to the patient's death), and by whom.
3. How frequently the EPaCCS record is updated once opened, and who makes any changes.
4. The underlying cause and place of death for EPaCCS and non-EPaCCS patients.
5. The number of hospital admissions and Emergency Department attendances for EPaCCS and non-EPaCCS patients in the last 12 months of life.

Descriptive data will be collected, by the CCG, on EPaCCS usage, including the number of records created by each GP surgery in the CCG area. Data from the integrated digital care record will be accessed to describe which HCPs (GPs, community nurses, hospice HCPs, ambulance HCPs and secondary care clinicians) are accessing these shared end-of-life care records.

For peer review only

Phase five: data analysis and synthesis of study findings (October 2018 – October 2019)

Quantitative methods

Quantitative data will be analysed using Stata v15 and reported using descriptive statistics. Within the context of this realist evaluation, we were keen to use the quantitative data to address a single hypothesis, namely whether nominal possession of an EPaCCS record was associated with increased chance of dying at home. However, logistic regression will be used to determine the adjusted odds ratio and 95% confidence intervals for the associations between having an EPaCCS record and dying at home, considering other factors of interest, including, but not limited to: age, sex, deprivation and underlying cause of death.

Of approximately 8,000 deaths occurring in the CCG area over the year of study, we expect around 10% (800 deaths) of patients to have an EPaCCS (10). If the proportion of deaths occurring at home is expected to be 25% among those without an EPaCCS, we would have over 99% power to detect an absolute increase of 10% to 35% among those with an EPaCCS. The power would be about 84% if the proportion were increased by 5% to 30%.

Descriptive statistics will be employed to report EPaCCS usage.

Qualitative methods

Data analysis will be conducted using a realist approach informed by Jackson and Kolla's realistic evaluation analysis method (29). This analytic process will involve the following steps:

1. Coding individual units (a discrete C, M or O) within the narratives of the interviews.
2. Identifying the complex connections that link these codes together into dyads or triads.
3. Subsuming the linked codes into themes using thematic analysis (30).

Analysis will begin shortly after data collection starts and be ongoing and iterative. Analysis will inform further data collection: for instance, analytic insights from data gathered in earlier interviews will be used to develop and adapt the programme theory and in turn, identify any changes that need to be made to the topic guide for use during later interviews. The study will generate new programme theories to explain how the EPaCCS works, for whom, and any contextual influences and constraining factors that affect their initiation and usage. Emerging analysis and findings will be discussed with PPI representatives, to explore and clarify findings.

Qualitative and quantitative data will be collected concurrently, giving equal weight to each (30). Data will be triangulated in order to test and refine the programme theories, accepting

For peer review only

that any findings are fallible and with time and further study new data are bound to emerge (31). The synthesised study findings will establish the potential outcomes of EPaCCS, identify the underlying mechanisms which explain how they produce these effects and highlight the key contextual factors that affect their success or failure. Recommendations can then be made for the development and implementation of EPaCCS.

Patient and Public Involvement

To support the development of this study protocol, members of the study team (LF and LP) presented and discussed an outline proposal of this study to patients, staff and carers at the local hospice on two separate occasions in April 2018. Approximately 10 participants voluntarily took part in these, semi-structured, discussions, in which we asked specific questions concerning ethical and methodological issues. Participants were also encouraged to ask any questions. These meetings raised several important issues which have been incorporated into the design of this study. Such issues included allowing patients the choice of whether to have a carer sit alongside them during their interview and which HCPs they felt it was important that the study team spoke to, due to the involvement they had in providing care for patients. The meetings also discussed what terms, wording and questions would be acceptable to patients and carers to read and hear in the study information documents and interviews.

At the end of both meetings, patients and carers were invited to continue to support the design of the study should they wished. Two members came forward expressing a wish to be more actively involved in the study. They have kindly been involved in reviewing all the study literature, including the topic guides, study information sheets and the lay summary for this protocol. It is hoped that they will wish to continue their involvement with the study. This will include informing the content of materials for lay audiences, drawing links to groups and forums that the research team may be unaware of, and supporting the study team

For peer review only

with the interpretation and dissemination of study findings. To ensure ongoing PPI, all patients and carers taking part in the study will be invited to support the ongoing development of the study.

Ethics and dissemination

The study has been approved by NHS South West – Frenchay Research Ethics Committee (REC reference number: 18/SW/0198).

The research team will disseminate the findings to a range of stakeholders. We will draw on the networks and expertise of the local CCG end-of-life care board to disseminate the research outputs widely and appropriately. Key audiences include patient and carer organisations, GPs and community nursing teams in primary care, ambulatory services and care home staff, HCPs working in secondary palliative care services and hospices, managers and directors within healthcare organisations with responsibility to provide high-quality services within budget and healthcare policymakers, nationally and internationally.

Acknowledgements

We would like to thank all the patients, carers and HCPs who helped to support the PPI in development of this study.

We would also like to thank Dr Justin Jagosh, Director of the Centre for Advancement in Realist Evaluation and Synthesis (https://realistmethodology-cares.org/), for his support and guidance in developing the initial programme theory.

Contributors

LP was the principal investigator for this qualitative study. LF, MF, RM and SP all contributed to the development of the study protocol. LP and LF drafted this manuscript. All

For peer review only

authors contributed to the editing of the final manuscript and the refining of its intellectual content.

Funding

LP's time is paid for by a NIHR School for Primary Care Research GP Career Progression Fellowship. This fellowship funding will also meet the research costs. LF's time is paid for by NIHR Research Capability Funding. MF's time is supported by the NIHR Collaboration for Leadership in Applied Health Research and Care West (NIHR CLAHRC West). The study is supported by the NIHR West of England Clinical Research Network. The views expressed in this publication are those of the authors and not necessarily those of the NHS, the National Institute for Health Research or the Department of Health and Social Care.

Competing interests

We have read and understood BMJ policy on declaration of interests and declare that we have no competing interests.

References

1. National Institute for Health and Care Excellence (2004). Improving supportive and palliative care for adults with cancer. Cancer service guideline [CSG4].
2. Palliative and end of life care Priority Setting Partnership: Putting patients, carers and clinicians at the heart of palliative and end of life care research. January 2015. Available at: https://www.mariecurie.org.uk/globalassets/media/documents/research/PeolcPSP_Final_Report.pdf (accessed 30/05/18)

For peer review only

3. Barker I, Steventon A, Deeny SR. Association between continuity of care in general practice and hospital admissions for ambulatory care sensitive conditions: cross sectional study of routinely collected, person level data. *BMJ* 2017;356:j84

4. Schweitzer B, Blankenstein N, Deliens L, Van Der Horst H. Out-of-hours palliative care provided by GP co-operatives in the Netherlands: A focus group study. *European Journal of General Practice*, 2011; 17: 160–166.

5. Hall S, Murchie P, Campbell C and Murray SA. Introducing an electronic Palliative Care Summary (ePCS) in Scotland: patient, carer and professional perspectives. *Family Practice* 2012; 29:576–585.

6. Richards SH, Winder R, Seamark C, Seamark D, Avery S. Gilbert J, Barwick A and Campbell JL. 2011: The experiences and needs of people seeking palliative health care out-of-hours: a qualitative study. *Primary Health Care Research & Development* 12(2), 165–78; available from: PM:21457601.

7. Leydon GM, Shergill NK, Campion-Smith C, et al. Discontinuity of care at end of life: a qualitative exploration of OOH end of life care. *BMJ Supportive & Palliative Care* 2013;3:412-421.

8. Purdy S, Lasseter G, Griffin T, Wye L. Impact of the Marie Curie Cancer Care Delivering Choice Programme in Somerset and North Somerset on place of death and hospital usage: a retrospective cohort study. *BMJ Supportive & Palliative Care* 2014;0:1–6.

9. Asprey A, Richards SH, Wright C, Seamark C, Seamark D and Moxon J. Transferring information to an out-of-hours primary care service for patients with palliative care needs: an action research study to improve the use of handover forms. *Primary Health Care Research & Development* 2013; 14: 7–20.

For peer review only

10. Wye L, Lasseter G, Simmonds B, Duncan L, Percival J and Purdy S. Electronic palliative care coordinating systems (EPaCCS) may not facilitate home deaths: A mixed methods evaluation of end of life care in two English counties. *Journal of Research in Nursing*, 2016, Vol. 21(2) 96–107

11. National Institute for Health and Clinical Excellence (2011) End of Life Care for Adults. NICE Quality Standard [QS13]

12. Petrova M, Riley J, Abel J and Barclay S. Crash course in EPaCCS (Electronic Palliative Care Coordination Systems): 8 years of successes and failures in patient data sharing to learn from. *BMJ Supportive & Palliative Care* 2016;0:1–9.

13. Ali AA, Adam R, Taylor D, et al. Use of a structured palliative care summary in patients with established cancer is associated with reduced hospital admissions by out-of-hours general practitioners in Grampian. BMJ Support Palliat Care2013;3:452–5.doi:10.1136/bmjspcare-2012-000371

14. Turner V, Flemming K. Socioeconomic factors affecting access to preferred place of death: A qualitative evidence synthesis. *Palliative medicine* 2019 Mar 8:0269216319835146.

15. Department of Health (2016) Our Commitment to you for end of life care: the Government Response to the Review of Choice in End of Life Care. Available at: https://assets.publishing.service.gov.uk/government/uploads/system/uploads/attachment_data/file/536326/choice-response.pdf (accessed 21/03/19)

16. Office for National Statistics. National Survey of Bereaved People (VOICES): England, 2015. Available at: https://www.ons.gov.uk/peoplepopulationandcommunity/healthandsocialcare/healthcaresystem/bulletins/nationalsurveyofbereavedpeoplevoices/england2015#preferences-and-choice-at-the-end-of-life (accessed 30/05/18)

17. Public Health England, End of Life Care Profiles. Available at: https://fingertips.phe.org.uk/profile/end-of-life (accessed 30/05/18)

18. Allsop MJ, Kite S, McDermott S, Penn N, Millares-Martin P and Bennett MI. Electronic palliative care coordination systems: Devising and testing a methodology for evaluating documentation, *Palliative Medicine* 2017 May; 31(5): 475–482.

19. Fallowfield L and Jenkins V. Communicating sad, bad, and difficult news in medicine. *The Lancet*, 2004;*363*(9405), 312-319.

20. Gibbins J, McCoubrie R, and Forbes K. Why are newly qualified doctors unprepared to care for patients at the end of life? *Medical education*;2011, *45*(4), 389-399.

21. Whole Systems Partnership. (2016) *Independent evaluation of Electronic Palliative Care Co-ordination Systems (EPaCCS) in England: Final Report*, March 2016

22. Wye L, Lasseter GM, Percival JF, Simmonds BAJ, Duncan LJ, Purdy S. Independent Evaluation of the Marie Curie Cancer Care Delivering Choice Programme in Somerset and North Somerset. Centre for Primary Health Care, Department of Social and Community Medicine, The University of Bristol, 2012.

23. NHS Digital (2015). SCCI1580: Palliative Care Co-ordination: Core Content. Available at: https://digital.nhs.uk/data-and-information/information-standards/information-standards-and-data-collections-including-extractions/publications-and-notifications/standards-and-collections/scci1580-palliative-care-co-ordination-core-content (accessed 23/01/19)

24. Pawson R and Tilley N. Realistic Evaluation. London: Sage, 2005.

25. National End of Life Care Intelligence Network. (2014). *Electronic Palliative Care Co-ordination Systems (EPaCCS) in England: Survey of clinical commissioning groups (2013).*

26. Pawson R. Evidence based policy: A realist perspective. London: Sage, 2006.

For peer review only

27. Greenhalgh T, Wong G, Jagosh J, et al Protocol—the RAMESES II study: developing guidance and reporting standards for realist evaluation BMJ Open 2015;5:e008567. doi: 10.1136/bmjopen-2015-008567

28. Sturges JE, Hanrahan KJ. Comparing telephone and face-to-face qualitative interviewing: a research note. Qual Res. 2004 Apr;4(1):107-18.

29. Jackson SF, Kolla G. A new realistic evaluation analysis method: linked coding of context, mechanism, and outcome relationships. American Journal of Evaluation. 2012 Sep;33(3):339-49.

30. Hanson WE, Creswell JW, Clark VL, Petska KS, Creswell JD. Mixed methods research designs in counseling psychology. Journal of counseling psychology. 2005 Apr;52(2):224.

31. Emmel N, Greenhalgh J, Manzano A, Monaghan M, Dalkin S (Eds). Doing realist research. London: Sage, 2018.

For peer review only

Figure 1 – Definition of Context, Mechanism and Outcome

Context

Something that existed prior to the introduction of the EPaCCS, for example cultural views and beliefs around talking about death and dying and Advanced Care Planning (ACP) conversations. Contexts might also refer to the setting, (e.g. primary or secondary care) or patient characteristics (e.g. underlying diagnosis, socio-demographics, mental capacity).

Mechanism

The intended or unintended resources created by an intervention and the response to those resources (cognitive, emotional, motivational etc) by Healthcare Professionals (HCPs), patients and carers. Mechanisms can pertain to why HCPs and patients choose (or choose not) to utilise the EPaCCS.

Outcome

An outcome will define the result of the EPaCCS whether intended (did the project succeed against the criteria it set itself at the outset), and also the unplanned and/or unexpected impacts.

Informed by (24) and (26).

For peer review only

Figure 2 – Visual representation of the initial programme theory at a macro level

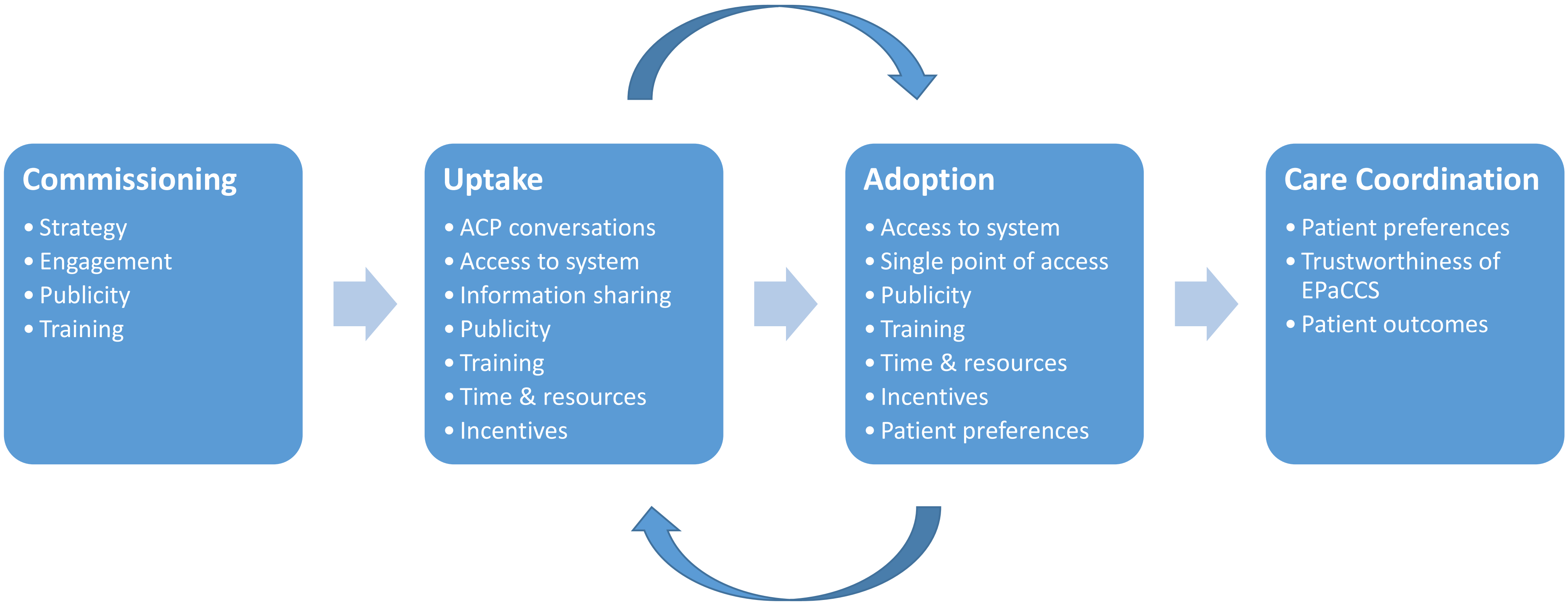

Supplement: Author's manuscript [file bmjopen-2019-031153.draft_revisions.pdf]
